# Gene expression profiling of Group 3 medulloblastomas defines a clinically tractable stratification based on *KIRREL2* expression

**DOI:** 10.1007/s00401-022-02460-1

**Published:** 2022-06-30

**Authors:** Andrey Korshunov, Konstantin Okonechnikov, Damian Stichel, Daniel Schrimpf, Alberto Delaidelli, Svenja Tonn, Martin Mynarek, Philipp Sievers, Felix Sahm, David T. W. Jones, Andreas von Deimling, Stefan M. Pfister, Marcel Kool

**Affiliations:** 1 Clinical Cooperation Unit Neuropathology (B300), German Cancer Research Center (DKFZ), Im Neuenheimer Feld 280, 69120 Heidelberg, Germany; 2 German Cancer Consortium (DKTK), Heidelberg, Germany; 3 Department of Neuropathology, Heidelberg University Hospital, Heidelberg, Germany; 4 Hopp Children’s Cancer Center Heidelberg (KiTZ), Heidelberg, Germany; 5 Division of Pediatric Neuro-Oncology (B062), German Cancer Research Center (DKFZ), Heidelberg, Germany; 6 Department of Molecular Oncology, British Columbia Cancer Research Centre, Vancouver, BC Canada; 7 Department of Pathology and Laboratory Medicine, British Columbia Cancer Research Centre, Vancouver, BC Canada; 8 Pediatric Hematology and Oncology, University Medical Center Hamburg-Eppendorf, Martinistr. 52, 20246 Hamburg, Germany; 9 Division of Pediatric Glioma Research (B360), German Cancer Research Center (DKFZ), Heidelberg, Germany; 10 Department of Pediatric Hematology and Oncology, Heidelberg University Hospital, Heidelberg, Germany; 11 Princess Máxima Center for Pediatric Oncology, 3584 CS Utrecht, The Netherlands

**Keywords:** Medulloblastoma, Group 3, *KIRREL2*, Expression, Prognosis

## Abstract

Medulloblastomas (MB) molecularly designated as Group 3 (Grp 3) MB represent a more clinically aggressive tumor variant which, as a group, displays heterogeneous molecular characteristics and disease outcomes. Reliable risk stratification of Grp 3 MB would allow for appropriate assignment of patients to aggressive treatment protocols and, vice versa, for sparing adverse effects of high-dose radio-chemotherapy in patients with standard or low-risk tumors. Here we performed RNA-based analysis on an international cohort of 179 molecularly designated Grp 3 MB treated with HIT protocols. We analyzed the clinical significance of differentially expressed genes, thereby developing optimal prognostic subdivision of this MB molecular group. We compared the transcriptome profiles of two Grp 3 MB subsets with various outcomes (76 died within the first 60 months vs. 103 survived this period) and identified 224 differentially expressed genes (DEG) between these two clinical groups (Limma R algorithm, adjusted *p*-value < 0.05). We selected the top six DEG overexpressed in the unfavorable cohort for further survival analysis and found that expression of all six genes strongly correlated with poor outcomes. However, only high expression of *KIRREL2* was identified as an independent molecular prognostic indicator of poor patients’ survival. Based on clinical and molecular patterns, four risk categories were outlined for Grp 3 MB patients: i. low-risk: M0-1/*MYC* non-amplified/*KIRREL2* low (*n* = 48; 5-year OS—95%); ii. standard-risk: M0-1/*MYC* non-amplified/*KIRREL2* high or M2-3/*MYC* non-amplified/*KIRREL2* low (*n* = 65; 5-year OS—70%); iii. high-risk: M2-3/*MYC* non-amplified/*KIRREL2* high (*n* = 36; 5-year OS—30%); iv. very high risk—all *MYC* amplified tumors (*n* = 30; 5-year OS—0%). Cross-validated survival models incorporating *KIRREL2* expression with clinical features allowed for the reclassification of up to 50% of Grp 3 MB patients into a more appropriate risk category. Finally, KIRREL2 immunopositivity was also identified as a predictive indicator of Grp 3 MB poor survival, thus suggesting its application as a possible prognostic marker in routine clinical settings. Our results indicate that integration of *KIRREL2* expression in risk stratification models may improve Grp 3 MB outcome prediction. Therefore, simple gene and/or protein expression analyses for this molecular marker could be easily adopted for Grp 3 MB prognostication and may help in assigning patients to optimal therapeutic approaches in prospective clinical trials.

**Supplementary Information:**

The online version contains supplementary material available at 10.1007/s00401-022-02460-1.

## Introduction

Medulloblastoma (MB) is a heterogenous disease comprised different clinico-molecular subtypes [3, 14, 15, 20, 22, 25, 28, 32]. In 2012, an international consensus on MB subgroups was reached among the pediatric neuro-oncology community, reporting four distinct “principal” molecular MB groups: WNT-MB, SHH-MB, Group 3 (Grp 3) MB and Group 4 (Grp 4) MB [20, 25, 32]. Since publication of this consensus, the biological and clinical relevance of these principal MB groups has been extensively reported, including methods for robustly assigning tumor samples to these variants based on either transcriptomic and/or methylome signatures [3, 15, 20, 21, 28–30]. Together, these advances recently culminated in the recognition of MB groups as part of the current 5th edition of the WHO Classification of CNS tumors [22], which recognizes four main molecular variants of the disease (WNT-MB, SHH-*TP53*^wild type^, SHH-*TP53*^mut^, and non-WNT/non-SHH). Namely the non-WNT/non-SHH MB cohort encompasses the Grp 3 MB and Grp 4 MB consensus molecular variants which together represent ~ 65% of all MB cases and have heterogeneous molecular characteristics and outcomes [3, 11, 13, 15, 20, 21, 28]. Numerous reports have indicated the presence of significant heterogeneity among Grp 3 MB, describing various and partly overlapping subgroups/subtypes differing both from clinical and molecular standpoints [3, 21, 28, 29]. While some of these Grp 3 MB are characterized by a favorable clinical course, other tumors are associated with extremely poor patient outcomes. In part, these latter are associated with high-risk clinico-molecular factors (young age, advanced M stage, large-cell/anaplastic histology (LCA), and *MYC* amplification) but substantial numbers of Grp 3 MB relapse in the absence of these risk indicators [3, 4, 7, 9, 21, 28].

Therefore, a more refined understanding of Grp 3 MB molecular heterogeneity is urgently needed for improved risk stratification, optimization of current treatments, and the development of subgroup-directed therapies. Studies investigating the clinical significance of various molecular features in Grp 3 MB at higher genomic resolution have recently been reported, identifying a wide range of prognostically relevant molecular patterns and subtypes [2, 3, 8, 9, 11, 15, 18–21, 24, 27–30]. Thus, combined multiple class-definition approaches to DNA-methylation profiles of Grp 3/Grp 4 MB identified eight second-generation subgroups, labeled I–VIII [21, 29]. Non-negative matrix factorization (NMF) analysis subdivided Grp 3 MB into high- and low-risk groups [28], whereas a similarity network fusion (SNF) analysis split Grp 3 MB into three clinically relevant subtypes [3]. It may be hypothesized that a variability of defined molecular prognosticators between these studies is a result of different study design, analytical parameter choice and cohort composition. Therefore, a method to resolve the inconsistencies between various outlined Grp 3 MB prognostic subtypes and/or markers is important for a consistent and unified risk stratification in the near future.

The objective of the current study is to identify clinically tractable molecular marker(s) to elaborate an optimal risk stratification of Grp 3 MB, suitable for application in routine clinical settings. For these purposes, we performed comparative gene expression RNA-based analysis of a representative Grp 3 MB patient cohort treated with risk adapted HIT-based protocols [4, 11]. We investigated the clinical significance of various genetic markers, developing an optimal prognostic subdivision of Grp 3 MB to prospectively assign upcoming tumor samples to clinically relevant risk subtypes.

## Materials and methods

### Patient population molecularly diagnosed Group 3 MB

A cohort of 179 pediatric MB diagnosed as “Grp 3 MB” with DNA methylation profiling (see below) was selected from the previously published international MB set molecularly analyzed at the German Cancer Research Centre [9, 15, 20, 23, 26, 30]. Informed written consent was obtained from all patients’ parents or other relatives/caregivers. This retrospective study was conducted under the auspices of the local Ethics Committees.

All 179 samples were classified as “Grp 3 MB” using the MNP v12.5 Random Forest classifier with a calibrated prediction score > 0.90 [21, 29]. The Grp 3 MB molecular group was confirmed using *t*-distributed stochastic neighbor embedding (t-SNE) and uniform manifold approximation and projection for dimension reduction (UMAP) methods as described [13, 16, 21]. In addition, the v12.5 Random Forest classifier also identified second-generation Grp 3 MB subgroups as described [21, 29].

All patients were uniformly treated within the period from 2001 to 2016. Treatment details and follow-up data were available for all patients who were operated and received combined treatments with HIT-based protocols as described [4, 11]. Briefly, three following regimens were applied: i. Chemotherapy (CHT) alone: HIT-SKK with intraventricular methotrexate injection for a part of infant patients (< 4 years) at any M stage (*n* = 39/22%). 2. Older patients with M0-1 at diagnosis received primary cranio-spinal irradiation (RT) in standard doses followed with maintenance chemotherapy (*n* = 42/23%). 3. Older patients at M2-3 stages received initially two cycles HIT-SKK chemotherapy followed by hyperfractioned RT and maintenance chemotherapy (*n* = 98/55%). Relapsed patients were managed with various modalities.

The follow-up analysis was frozen on 01.01.2022 as the end-point and the median time of observation was 82 months. Progression-free survival (PFS) was calculated from the date of diagnosis until tumor recurrence or last contact for patients who were free of disease. Overall survival (OS) was calculated from the date of diagnosis until death of patient from disease or last contact for patients who were still alive.

### RNA sequencing analysis

RNA was extracted from formalin-fixed and paraffin-embedded (FFPE) tissue samples and RNA sequencing was performed on a NextSeq 500 (Illumina) as described [16]. The reads were aligned to hg19 reference using STAR version 2.5.2b and for each sample, gene expression was quantified by the feature counts module of the Subread package version 1.4.6 using Gencode version 19 annotations with considering uniquely mapped reads only. Tumor sample comparisons were performed with *log2* RPKM expression normalization [16]. Differential gene expression analysis between clinical groups was performed by comparing one molecular class against the other using Limma R package (adjusted *p*-value < 0.05). Gene ontology analysis was done using ClueGO with visualization using Cytoscape version 3.4. [16]. For survival analyses based on identified differentially expressed genes, samples were categorized as having high and low mRNA levels using a cut-off in expression that resulted in the lowest log-rank *p*-value using a Bonferroni correction for multiple testing [33].

### Fluorescence in situ hybridization (FISH)

Multicolor interphase fluorescence in situ hybridization (FISH) analysis for *MYC* (8q24) was performed for all 179 Grp3 MB samples as described [23].

### Immunohistochemistry (IHC) With KIRREL2 Antibody

IHC was conducted on 4-µm thick FFPE tissue sections mounted on adhesive slides followed by drying at 80 °C for 15 min. For IHC analysis, a rabbit polyclonal KIRREL2 antibody (rabbit polyclonal; PA5-72823, Invitrogen) was applied. IHC was performed with an automated immunostainer (Benchmark; Ventana XT) using antigen-retrieval protocol CC1 and a working antibody dilution of 1:100 for KIRREL2 with incubation at 37 °C for 32 min.

### Statistics

The distributions of progression-free survival (PFS) and overall survival (OS) were calculated according to the Kaplan–Meier method using the log-rank test. For multivariate analysis, Cox proportional hazards regression models were used and estimated hazard ratios are provided with 95% confidence intervals. The ability of Cox models to classify risk was assessed by computing the area under the time-dependent receiver operating characteristic (ROC) curves, calculated according to the Nearest Neighbor Estimation (NNE) method. ROC curves were computed every 18 months of follow-up time up to 10 years, and the resulting areas under the curve were compared by paired *t*-test. Risk categories were defined as follows: low risk, 10-year survival ≥probability ≥ 0.9; standard-risk, 10-year survival probability ≥ 0.75 and < 0.9; high-risk, 10-year survival probability ≥ 0.5 and 0.75; very high-risk, 10-year survival probability < 0.5. Statistical analyses were performed with R 3.5.1, with packages “survival’, “survminer” and “maxstat” for uni and multivariate survival analyses, “pec” and “survivalROC” for prediction error and ROC curves.

## Results

### Clinical and molecular characteristics of Grp 3 MB cohort and second-generation subgroups

Clinical and molecular characteristics of 179 Grp 3 MB patients are summarized in Table 1. Patients were aged between 0 and 16 years (median: 5.4), with a preponderance of male patients (male:female ratio = 2.5:1). More than half of the patients (98/55%) revealed M2-3 stages at initial presentation. Disease relapses were identified in 98 patients (55%; in a vast majority (95%)—as metastatic dissemination), and 78 of them (78% of relapsed patients) succumbed to their disease. Of those, 76 patients (98%) died within the first 60 months after operation.

**Table 1:** Clinico-pathological variables of Group 3 MB and second-generation subgroups

Variables	Grp 3 MB (179)	SG II (70)	SG III (38)	SG IV (42)	SG V (14)	SG VII (15)
Age median	5.6	6.1	5.7	3.2	5.8	6.7
Age: infants/children	35%/65%	20%/80%	15%/85%	70%/30%	10%/90%	10%/90%
Gender: male/female	70%/30%	70%/30%	80%/20%	55%/45%	70%/30%	70%/30%
M stage: M0-1/M2-3	45%/55%	40%/60%	50%/50%	40%/60%	45%/55%	65%35%
Histology: classic/LCA	65%/35%	50%/50%	80%/20%	65%/35%	75%/25%	90%/10%
RT + CHT/CHT alone	80%/20%	80%/20%	90%/10%	60%/40%	95%/5%	95%/5%
5-year PFS	50%	45%	40%	55%	45%	75%
5-year OS	60%	50%	45%	65%	65%	85%
Amplifications	25%	45%	20%	10%	50%	0
*MYC* amplification	20%	40%	10%	5%	15%	0
*MYCN* amplification	5%	2%	0	5%	30%	0
*CCND2* amplification	10%	0	0	0	15%	0
1q gain	25%	50%	20%	40%	50%	10%
Trisomy 7	45%	20%	50%	65%	40%	75%
Monosomy 8	25%	5%	20%	40%	20%	60%
8q gain	30%	60%	20%	5%	10%	5%
10q loss	45%	20%	70%	50%	50%	50%
Monosomy 11	30%	15%	15%	50%	15%	30%
12q gain	25%	15%	15%	40%	40%	10%
16q loss	45%	35%	45%	50%	70%	30%
i(17q)	35%	40%	55%	15%	90%	10%
Trisomy 17	20%	25%	15%	50%	10%	55%
18q gain	30%	10%	10%	60%	20%	50%

*SG* second-generation subgroup

Amplification of *MYC* oncogene was detected in 30 Grp 3 MB (20%) and confirmed by FISH analysis in all samples. Among frequent copy number variants (CNVs) were trisomy 7 (45%), 10q loss (45%), 16q loss (45%), and i(17q) (35%) (Table 1).

By univariate survival analysis, advanced M2-3 stages, LCA histology, *MYC* amplification, and i(17q) were associated with poor survival, whereas RT, trisomy 7, monosomy 8, and gain 18q were defined as favorable prognosticators for Grp 3 MB (Table 2). Advanced M stages, applied RT, and *MYC* amplification were independent variables in a Cox regression model.

**Table 2:** Uni- and multivariate survival analyses for Group 3 MB cohort

Variables Group 3	HR U PFS	*p*-value	HR M PFS	*p*-value	HR U OS	*p*-value	HR M OS	*p*-value
M stage: M2-3 vs. M0-1	13.26	< 0.01	2.71	0.04	19.65	< 0.01	3.28	< 0.01
Histology: LCA vs. Classic	11.58	< 0.01	–	–	14.51	< 0.01	–	–
CHT alone vs. RT + CHT	44.91	< 0.01	4.31	< 0.01	39.31	< 0.01	3.25	< 0.01
Amplifications: yes vs. no	22.95	< 0.01	–	–	22.79	< 0.01	–	–
*MYC* amplification: yes vs. no	49.32	< 0.01	3.83	< 0.01	64.61	< 0.01	4.34	< 0.01
Trisomy 7: yes vs. no	0.47	0.04	–	–	0.38	< 0.01	–	–
Monosomy 8: yes vs. no	0.35	< 0.01	–	–	0.46	0.03	–	–
i(17q): yes vs. no	11.87	< 0.01	–	–	14.67	< 0.01	–	–
18q gain: yes vs. no	0.38	< 0.01	–	–	0.37	< 0.01	–	–
Subgroup: II/III/V vs. IV/VII	5.36	0.02	–	–	7.91	< 0.01	–	–
*MYC* expression*:* high vs. low	53.14	< 0.01	–	–	67.41	< 0.01	–	–
*KIRREL2* expression: high vs. low	73.41	< 0.01	7.38	< 0.01	78.71	< 0.01	8.87	< 0.01
*ITPRL1* expression: high vs. low	40.51	< 0.01	–	–	47.45	< 0.01	–	–
*DCAF4* expression: high vs. low	30.69	< 0.01	–	–	40.95	< 0.01	–	–
*CTD1* expression: high vs. low	37.74	< 0.01	–	–	57.58	< 0.01	–	–
*NPW* expression: high vs. low	40.21	< 0.01	–	–	58.61	< 0.01	–	–

*LCA* large-cell anaplastic, *CHT* chemotherapy, *RT* radiotherapy, *HR U* hazard ration univariate analysis, *HR M* hazard ration multivariate model, *PFS* progression-free survival, *OS* overall survival, *p-value* log-rank test

Five second-generation subgroups were outlined within Grp 3 MB by methylation analysis [21, 29]. Among them were subgroups II (*n* = 70; 40%), III (*n* = 38; 21%), IV (*n* = 42; 23%), V (*n* = 14; 8%) and VII (*n* = 15; 8%). We did not find Grp 3 MB with molecular subgroup signatures I, VI and VIII in this cohort. In line with previous studies [21, 29], different second-generation Grp 3 MB revealed subgroup-specific clinical variables and molecular aberrations (Table 1; Online Resource Supplementary Fig. 1). Thus, *MYC* amplification/8q gain were frequent in subgroup II, whereas monosomy 8 was mostly identified in subgroups IV and VII. Isochromosome 17q was frequent in subgroup V, but trisomy 17 was identified in subgroups IV and VII. Clinically, subgroups II, III and V were associated with unfavorable PFS and OS as compared to subgroups IV and VII (Online Resource Supplementary Fig. 2).

### Genes differentially expressed between Grp3 MB with various clinical outcomes

**Fig. 1:**
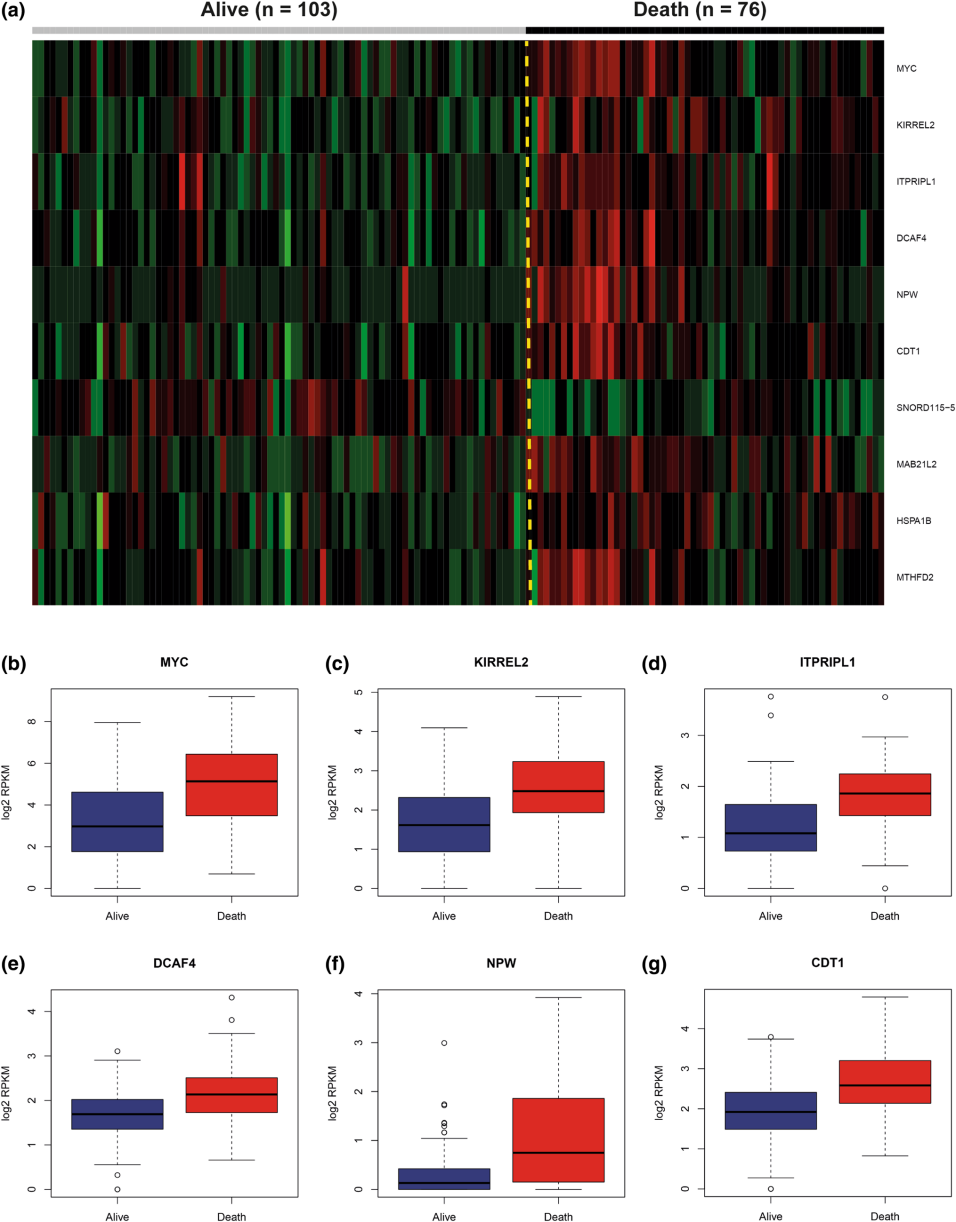
**a** Heat-map generated for a set of the top most-confident genes differentially expressed between survivors (*n* = 103) and non-survivors (*n* = 76) in Grp 3 MB**. b–g** Box plots for 6 top genes differentially expressed between survivors (Alive; blue boxplots) and non-survivors (Death; red boxplots); Among them are *MYC* (**b**); *KIRREL2* (**c**); *ITPRIPL1* (**d**); *DCAF4* (**e**); *NPW* (**f**); *CDT1* (**g**); (all Limma algorithm; *p* < 0.01)

We compared gene expression profiles generated by RNA sequencing between the two following clinical cohorts of Grp 3 MB patients: (i). those who died within the first 60 months after diagnosis (*n* = 76), and (ii). those who survived this period of time (*n* = 103). Using Limma R algorithm (see “Materials and methods”), we detected 224 genes and processed pseudogenes differentially expressed (DEG) between these two Grp 3 MB clinical subsets, with *MYC* on the top of this list (Fig. 1a; Online Resource Supplementary Table 1). In total, 154 DEG were highly expressed in the “poor survival” subset; among them prevailed genes of the ribosomal protein family L/S (*RPL/RPS*; *n* = 25), histone family (*HIST*; *n* = 12), and eukaryotic initiation factor family (*EIF*; *n* = 6). In contrast, 70 genes were overexpressed in the subset with favorable outcomes; among them solute carrier family genes (*SLC*; *n* = 6), protocadherin family genes (*PCDH*; *n* = 5), and small nucleolar family genes (*SNORD*; *n* = 5) were frequent.

**Fig. 2:**
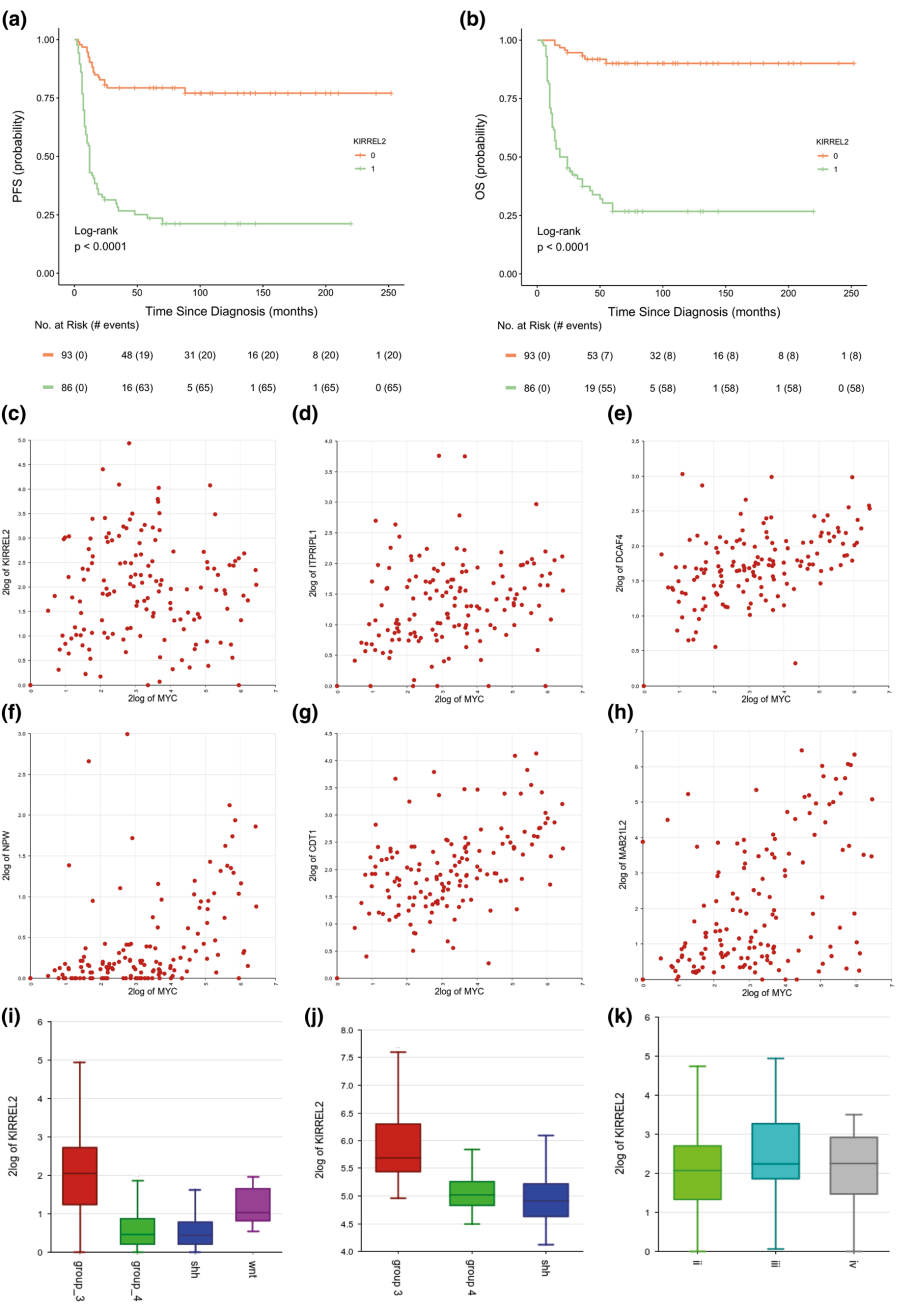
Progression-free (**a**) and overall (**b**) survival analysis revealed that high levels of *KIRREL2* expression (a cut-off *log2* RPKM > 2.5; green line) are significantly associated with worse outcomes in Grp 3 MB (log-rank test; *p* < 0.01). **c–h** Scatter graphs show an absence of correlation between *KIRREL2* and *MYC* expression (**c**; correlation coefficient: − 0.074; *p *= 0.374), whereas expression of *ITPRIPL1* (**d**)*, DCAF4* (**e**)*, NPW* (**f**)*, CDT1* (**g**)*, MAB21L2* (**h**) was correlated strongly with *MYC* expression (all p < 0.01). **i.** *KIRREL2* expression was higher in Grp 3 MB (red boxplot; *n* = 179) as compared to WNT-MB (violet boxplot; *n* = 20), SHH-MB (blue boxplot; *n* = 188) and Grp 4 MB (green boxplot; *n* = 260) in the current/screening RNA_seq MB set (*t*-test; *p* < 0.01). **j** *KIRREL2* expression was also higher in Grp 3 MB (red boxplot; *n* = 46) as compared to SHH-MB (blue boxplot; *n* = 51) and Grp 4 MB (green boxplot; *n* = 188) in independent/validation set generated with Affymetrix platform (*t*-test; *p* < 0.01). **k.** *KIRREL2* expression is quite similar in these main second generation II (light-green boxplot), III (dark-green boxplot) and IV (gray boxplot) subgroups which composed 85% of Grp 3 MB (*t*-test; *p* = 0.323)

Next, we analyzed the clinical relevance for six top DEG (*MYC, KIRREL2, ITPRIPL1, DCAF4, NPW, CTD1;* Fig. 1b–g). Univariate survival analysis showed that high expression levels (see “Materials and methods”) were associated with unfavorable clinical Grp 3 MB outcomes (Table 2). However, Cox regression models generated for Grp 3 MB (accounting for clinical and molecular data) disclosed that only high level of *KIRREL2* expression (with a cut-off level = 2.5 *log2* RPKM) was independently associated with adverse outcomes irrespective of other clinico-molecular variables (Fig. 2a, b; Table 2). A high level of *KIRREL2* expression was also associated with unfavorable survival for all second-generation subgroups from II to VII (not shown). However, we did not find any clinical significance for genes which were previously reported as molecular Grp 3 MB prognosticators [2, 8, 9, 18, 19, 24, 27, 33] but were not included in the current DEG set (Online Resource Supplementary Table 2).

In line with its independent clinical significance (Fig. 2c; Online Resource Supplementary Table 3), expression of *KIRREL2* was not correlated with *MYC* expression (correlation coefficient = 0.074; *p* = 0.373), whereas mRNA levels for other 5 top “non-*MYC*” DEG were correlated strongly with *MYC* expression (Fig. 2d–h; *p* < 0.01 for all). In turn, *KIRREL2* was positively correlated with a set of 60 genes involved in signaling pathways associated with ligase activity, tRNA metabolism, mitochondrial translation and neurogenesis (Online Resource Supplementary Tables 4 and 5). Only five of these *KIRREL2*-correlated genes (*GSG1, NTN3, EML4, PDIA5, LHX3*) were overexpressed in the DEG set associated with unfavorable Grp 3 MB outcomes.

Also, top six clinically relevant DEG disclosed variable expression within different molecular MB groups and second-generation subgroups (Online Resource Supplementary Figs. 3 and 4). However, only *KIRREL2* was differentially expressed between Grp 3 and other MB groups in both the current-screening (Fig. 2i) and independent-validation tumor cohorts (Fig. 2j). No significant difference in expression was seen between second-generation subgroups II, III and IV, which comprised a vast majority of Grp 3 MB in this cohort (Fig. 2 k). We did not find survival differences related to various *KIRREL2* expression within SHH-MB (*n* = 188) and Grp 4 MB (*n* = 260) (data not shown).

DNA copy number status at the *KIRREL2* location (19q13.12) did not differ significantly between Grp3 MB with high and low gene expression levels, respectively. However, comparing *KIRREL2* expression and epigenetic data overlapping gene promoter region (1500 bp upstream) we identified that methylation levels of three CpG sites (cg15509065, cg21057435, and cg23087300) were significantly lower in Grp 3 MB samples with high *KIRREL2* expression. Also, negative correlation between gene expression and three CpGs methylation levels was identified (correlation coefficient *r* = − 0.413; − 0.309; and − 0.485, respectively; Online Resource Supplementary Table 6, Fig. 5). Moreover, low methylation levels for CpG cg15509065 and cg23087300 (with a cut-off level = 0.35) were associated with unfavorable Grp 3 MB survival but did not reach an independent level in the Cox regression model (data not shown).

Survival analyses of two public gene expression data generated with the Affymetrix platform for independent multi-institutional MB cohorts [3, 15] also showed unfavorable outcomes for Grp 3 MB with high *KIRREL2* expression, thus confirming data obtained with our RNA sequencing analysis (Online Resource Supplementary Fig. 6).

### The development of biomarker-driven risk stratification of Grp3 MB.

**Fig. 3:**
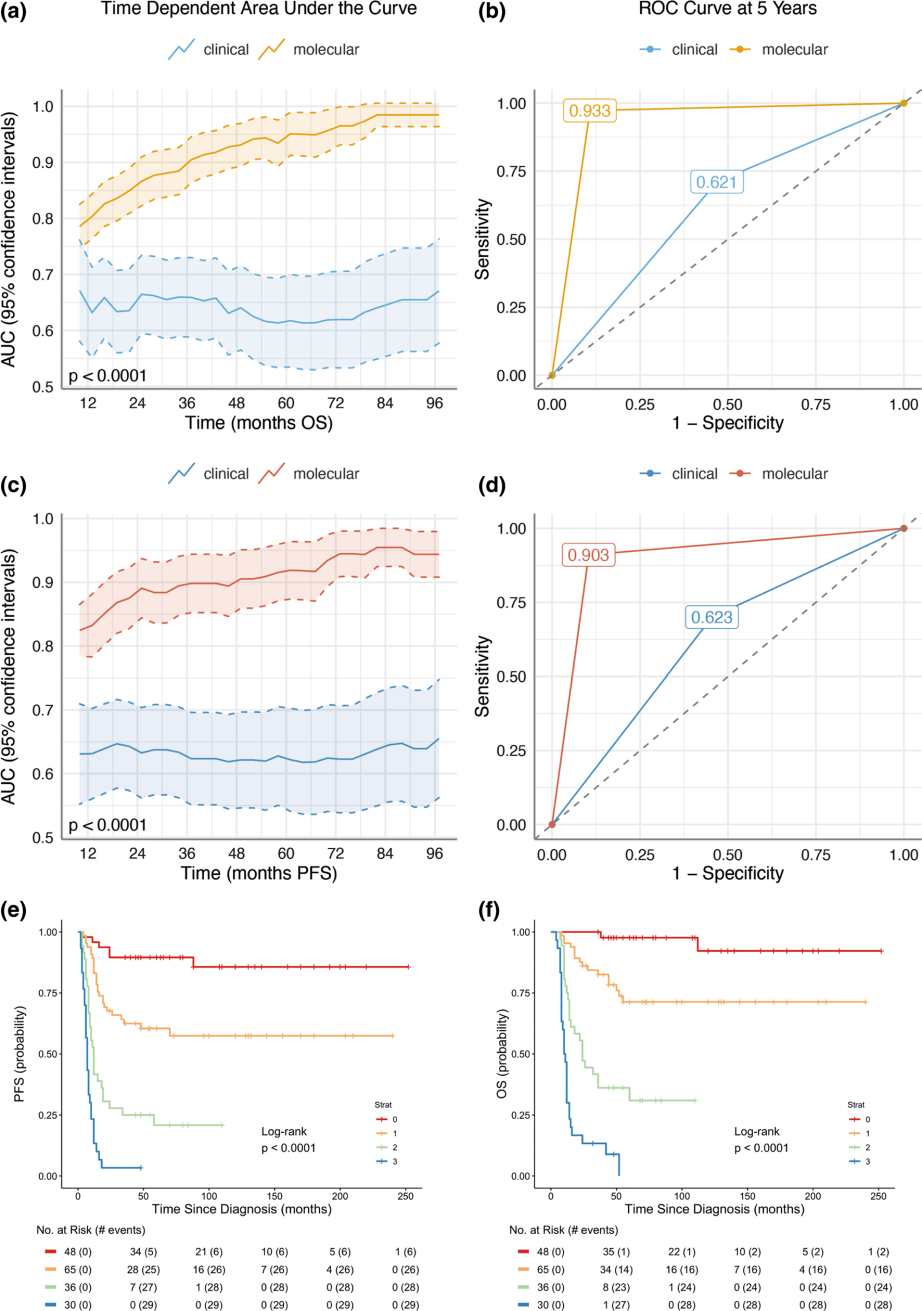
Area under the time-dependent receiver operating characteristic (AUC and ROC) curves for Grp 3 MB disease outcome (**a**, **b**) and progression (**c**, **d**) applying clinical variables alone (CSI RT and M stage blue line for PFS and OS), and the molecular marker *KIRREL2* (gold line for OS and red line for PFS). Thus, inclusion of *KIRREL2* expression in current stratification model significantly improves outcome prediction and reduced prediction error. For Grp 3 MB, four risk categories were outlined in terms of PFS (**e**) and OS (**f**): i. low-risk (line 0): M0-1/*MYC* non-amplified/*KIRREL2* low (*n* = 48; OS—95%); ii. standard-risk (line 1): M0-1/*MYC* non-amplified/*KIRREL2* high or M2-3/*MYC* non-amplified/*KIRREL2* low (*n* = 65; OS—70%); iii. high-risk (line 2): M2-3/*MYC* non-amplified/ *KIRREL2* high (*n* = 36; OS—30%); iv. very high risk (line 3)—all *MYC* amplified tumors (*n* = 30; OS—0%)

Accordingly, we selected *KIRREL2* as an optimal candidate and compared stratification regression models with and without information on gene expression. Inclusion of *KIRREL2* significantly improved outcome prediction for the current Grp 3 MB cohort, reducing prediction errors. Similar results were obtained when we compared receiver areas under curves (AUC) and operating characteristic curves (ROC) for the Cox models at different time points (Fig. 3a–d). To further underscore the prognostic relevance of this genomic marker, we combined the conventional risk variables with expression of *KIRREL2*. Inclusion of this molecular variable resulted in improvement of a stratification model identifying four risk categories for Grp 3 MB (Fig. 3e, f; Table 3): i. low-risk: M0-1/*MYC* non-amplified/*KIRREL2* low (*n* = 48; 5-year OS—95%); ii. standard-risk: M0-1/*MYC* non-amplified/*KIRREL2* high or M2-3/*MYC* non-amplified/*KIRREL2* low (*n* = 65; 5-year OS—70%); iii. high-risk: M2-3/*MYC* non-amplified/*KIRREL2* high (*n* = 36; 5-year OS—30%); iv. very high-risk—all *MYC* amplified tumors (*n* = 30; 5-year OS—0%). Grp 3 MB allocated to second-generation subgroups were evenly distributed between these four risk groups, excluding subgroup II which was frequent in the “very high-risk” subset and subgroup VII which was found mostly in the “low-risk” cohort (Table 3).

**Table 3:** Clinico-molecular characteristics within 4 outlined risk subsets within Group 3 MB

Variables	Low-risk (48/26%)	Standard-risk (65/40%)	High-risk (36/17%)	Very high-risk (30/17%)
Age (median)	6.8	5.3	4.7	5.1
Age: infant/children	20%/80%	30%/70%	40%/60%	40%/60%
Gender: male vs. female	80%/20%	65%/35%	75%/25%	60%/40%
M stage: M0-1/ M2-3	100%/0	65%/35%	0/100%	5%/95%
Histology: classic vs. LCA	75%/25%	70%/30%	55%/45%	5%/95%
RT + CHT vs. CHT alone	85%/15%	80%/20%	65%/35%	60%/40%
Recurrence	15%	45%	75%	95%
5-year PFS	85%	65%	20%	0
Death	5%	25%	65%	95%
5-year OS	95%	75%	30%	0
SG Subgroup II	35%	25%	25%	80%
SG Subgroup III	15%	25%	35%	10%
SG Subgroup IV	15%	30%	35%	5%
SG Subgroup V	5%	10%	5%	5%
SG Subgroup VII	30%	10%	0	0
Amplifications	5%	15%	10%	100%
*MYC* amplification	0	0	0	100%
1q gain	15%	15%	25%	50%
Trisomy 7	55%	50%	55%	10%
Monosomy 8	40%	40%	20%	10%
8q gain	30%	25%	25%	100%
10q loss	40%	50%	75%	50%
Monosomy 11	30%	25%	50%	0
16q loss	50%	50%	50%	15%
i(17q)	30%	30%	40%	70%
18q gain	30%	25%	25%	10%

*LCA* large-cell/anaplastic MB, *RT* radiotherapy, *CHT* chemotherapy, *PFS* progression-free survival, *OS* overall survival, *SG* second-generation subgroup

**Fig. 4:**
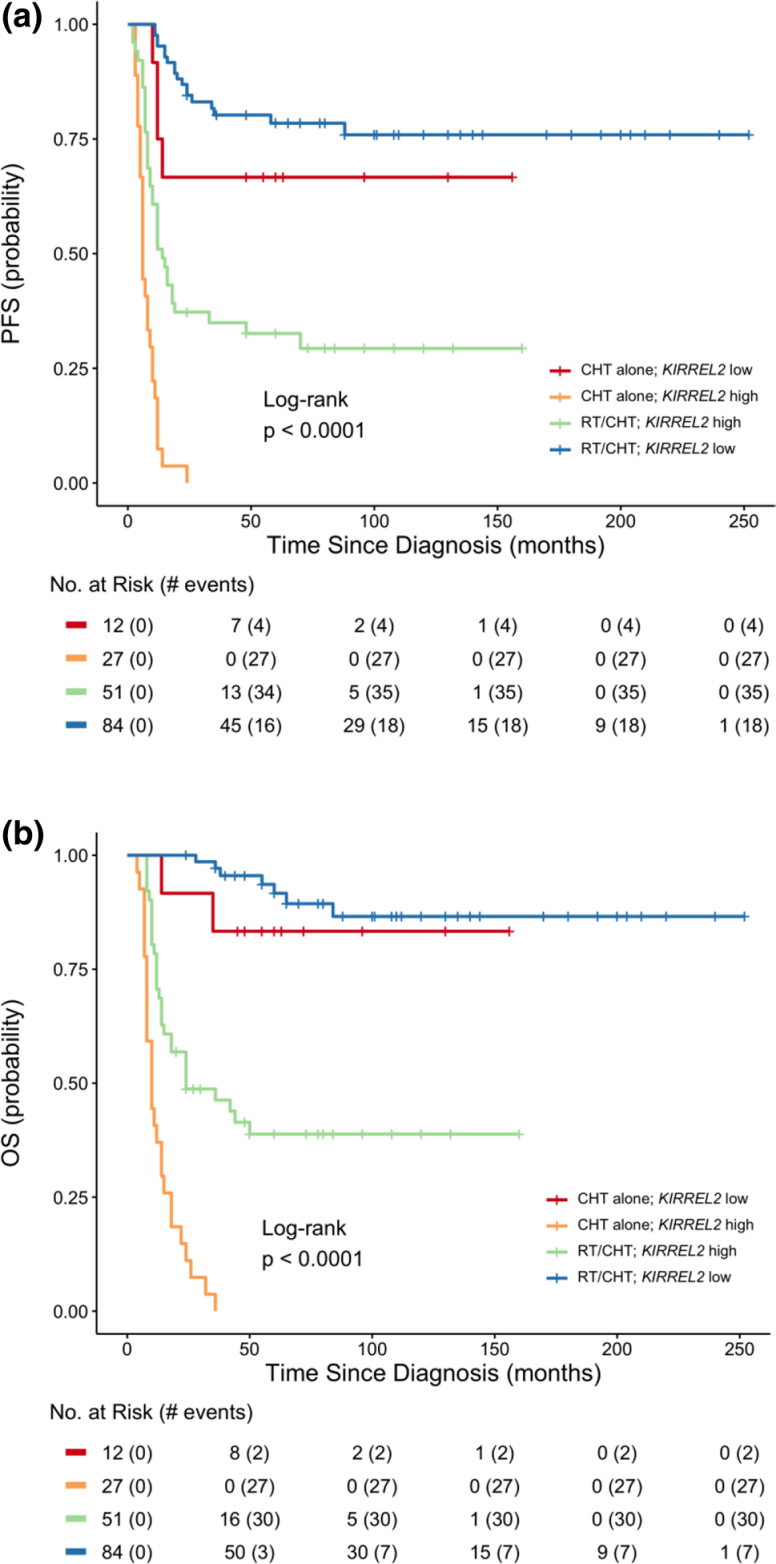
Progression-free (**a**) and overall (**b**) survival for Grp 3 MB combining various HIT regimens (RT/CHT vs. CHT alone) and *KIRREL2* expression levels. High *KIRREL2* was associated with adverse outcomes for patients treated either with CHT alone or combined RT/CHT as compared to those with low levels of *KIRREL2*

Importantly, approximately 60% of molecularly identified low- or standard-risk Grp 3 MB according to the current model were classified as high-risk according to conventional clinical standards, and, vice versa*,* 15% of clinically low-risk MB were reclassified as high-risk by the proposed stratification model. Additionally, in Grp 3 MB, high *KIRREL2* was associated with adverse outcomes for patients treated either with CHT alone or combined RT/CHT as compared to those with low levels of *KIRREL2* (Fig. 4a, b).

### IHC with KIRREL2 a possible tool for of Grp 3 MB prognostication

**Fig. 5:**
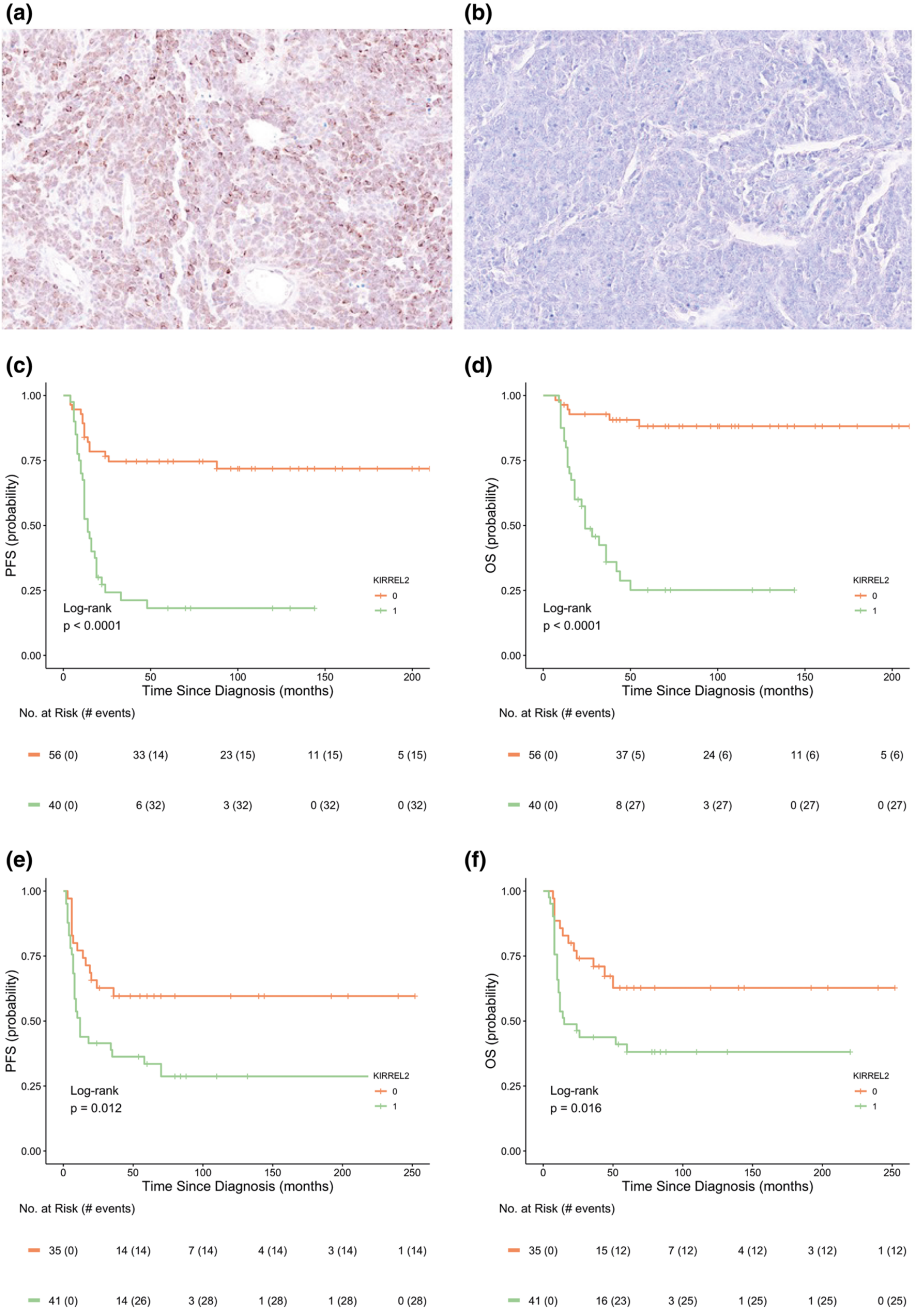
**a** Diffuse KIRREL2 membranous-cytoplasmic immuno-expression in Grp 3 MB accompanied with high gene expression at mRNA level. **b** Completely KIRREL2-negative Grp3 MB sample with low gene expression at mRNA level. Progression-free (left) and overall (right) survival analysis revealed that KIRREL2 immunopositivity (green line) is significantly associated with worse outcomes in both screening/whole sections (**c**, **d**) and validation/TMA sections (**e**, **f**) sets of Grp 3 MB (log-rank test; *p* < 0.01)

We applied KIRREL2 antibody to stain 96 Grp 3 MB with accessible whole tumor sections from the current transcriptome analysis cohort (screening set) and 76 samples from multi-institutional molecularly diagnosed Grp 3 MB cohort constructed on TMA sections and applied in previous study (independent/validation set) [15, 20]. The two following patterns of membranous-cytoplasmic KIRREL2 immunostaining were detected: (i) “Positive”—tumor sample was either patchily or entirely/diffusely stained (Fig. 5a). (ii) “Negative”—no IHC expression was found throughout the entire sample. (Fig. 5b). Three investigators showed perfect interobserver agreement for this categorization (*κ* = 1), and we did not find differences in terms of staining intensity across both tumor sets. In the screening set, KIRREL2 expression data coincided strongly between mRNA and protein levels (correlation coefficient *r* = 0.973; *p* < 0.01). Survival analysis revealed that KIRREL2 immunopositivity is significantly associated with worse outcomes in both the screening and validation sets of Grp 3 MB (Fig. 5d, e) by uni- and multivariate analyses. Thus, the results of KIRREL2 IHC prognostic evaluation corroborated with the survival data obtained by transcriptome analysis. In turn, 72 SHH-MB samples and 87 Group 4 MB samples were all completely immunonegative for KIRREL2.

## Discussion

The development of an optimal risk stratification for Grp 3 MB patients using molecular tools available in a routine and reproducible setting is important for clinical trial design. Rapid and reliable identification of high-risk Grp 3 MB would allow for assigning patients to appropriate aggressive treatment protocols and, vice versa, to sparing adverse effects of high-dose radio-chemotherapy for patients with standard or low-risk tumors. Although high-throughput DNA- and/or RNA-based prognostic stratification schemes undoubtedly proved their clinical significance [3, 21, 28], these advanced molecular techniques for Grp 3 MB risk subdivision are for the most part not available for routine practice world-wide. Therefore, bridging the gap between Grp 3 MB “next generation” molecular stratification and clinical practice, we tried to identify reliable genomic marker(s) that can be applied with inexpensive, accessible, and efficient method(s) for the identification of tumor risk categories. Comparing transcriptome profiles generated for Grp 3 MB survivors and non-survivors, we detected a set of 224 differentially expressed genes that were also identified as strong predictors of unfavorable behavior. *KIRREL2* overexpression was identified as the strongest prognostic indicator, confirmed in clinico-molecular multivariate models. Notably, *KIRREL2* expression differed strongly between various MB molecular groups, but was evenly distributed within three main Grp 3 MB second-generation subgroups, and its expression was independent of *MYC* alterations.

The *KIRREL2* gene encodes a type I transmembrane protein KIRREL2/NEPH3 which is a member of the immunoglobulin superfamily of cell adhesion molecules [17]. This protein also localizes to adherent junctions in pancreatic beta cells and may play a role in glomerular development in kidney. Gene function of *KIRREL2* is associated with negative regulation of cellular metabolic processes. Overexpression of *KIRREL2* at mRNA and/or protein level has been closely associated to advanced tumors stages, metastases development, drug resistance and poor prognosis of human cancers, although the underlying molecular mechanisms are still unclear [12, 17]. Mutations of *KIRREL2* were not identified in Grp 3 MB [21] and gene expression level was not associated with focal CNVs at the *KIRREL2* chromosomal localization. However, methylation levels of three CpGs within *KIRREL2* promoter region were negatively correlated with gene expression levels. Therefore, we suggest that the clinically relevant transcriptional diversity of *KIRREL2* in Grp 3 MB is reliably associated with CpGs methylation within the gene promoter region and, respectively, might be driven by molecular mechanisms associated with epigenetic dysregulation.

Additionally, *KIRREL2* is selectively expressed in developing CNS regions including the cerebellum and these cerebellar KIRREL2-positive cells appeared to be proliferative neural progenitors which have the potency to generate Purkinje cells [2, 34]. Therefore, *KIRREL2* overexpression in some Grp 3 MB may suggest their biological resemblance to mitotically active and poorly differentiated cerebellar neural progenitors thus partly explaining the clinical aggressiveness of this tumor subtype.

Because *KIRREL2* expression was the strong independent indicator of Grp 3 MB poor prognosis, a risk stratification model combining this molecular pattern with M-stage and *MYC* amplification (detected, in turn, either with FISH, CISH or DNA methylation array) may act as a useful tool for further routine application. Moreover, *KIRREL2* expression was also predictive of poor response to various treatment modalities (with and without RT), thus underscoring its potential usefulness not only in Grp 3 MB prognostication but also for therapy assignment, although this assertion needs to be confirmed within prospective randomized clinical trials. Risk stratification and accurate outcome prediction of future Grp 3 MB cohorts in the absence of high-throughput profiling techniques may be enhanced by assessing *KIRREL2* expression in routine neuropathology. For example, single gene RQ-PCR quantification, Taqman low-density arrays, or Nanostring-based analyses evaluating expression of this gene might be easily developed in neuropathological practice after elaboration of optimal cut-off levels for each method applied [1, 5, 6, 10, 14, 31]. In addition, KIRREL2 protein expression was defined here as a prognostic indicator. Therefore, KIRREL2 immunohistochemistry may also be considered as a potent marker for further Grp 3 MB stratification. Moreover, because KIRREL2 immuno-expression was not identified in SHH-MB and Grp 4 MB, a utility of this marker may not require an identification of molecular MB group before its prognostic implication could have relevance in clinical settings.

In summary, current results indicate that integration of *KIRREL2* expression in risk stratification models may improve Grp 3 MB outcome prediction. It has important clinical relevance, as a simple expression analysis for this predictive molecular marker at mRNA or protein level could be adopted in neuropathology laboratories world-wide. Rapid risk stratification of Grp 3 MB combining clinical and molecular patterns will help in assigning these patients into individual treatment protocols. Future works should aim at validating the relevance of the proposed Grp3 MB stratification approach in prospective clinical trials.

## Supplementary Information

Below is the link to the electronic supplementary material.

Supplementary file1 (PDF 872 kb)
