# Supplementary material for: Gene expression profiling of Group 3 medulloblastomas defines a clinically tractable stratification based on KIRREL2 expression
Source: Acta Neuropathol. 2022 Jun 30;144(2):339–52. doi: 10.1007/s00401-022-02460-1 (PMC9288368; doi:10.1007/s00401-022-02460-1)
Supplement: Supplementary file 1 — Supplementary file1 (PDF 872 kb) [file 401_2022_2460_MOESM1_ESM.pdf]

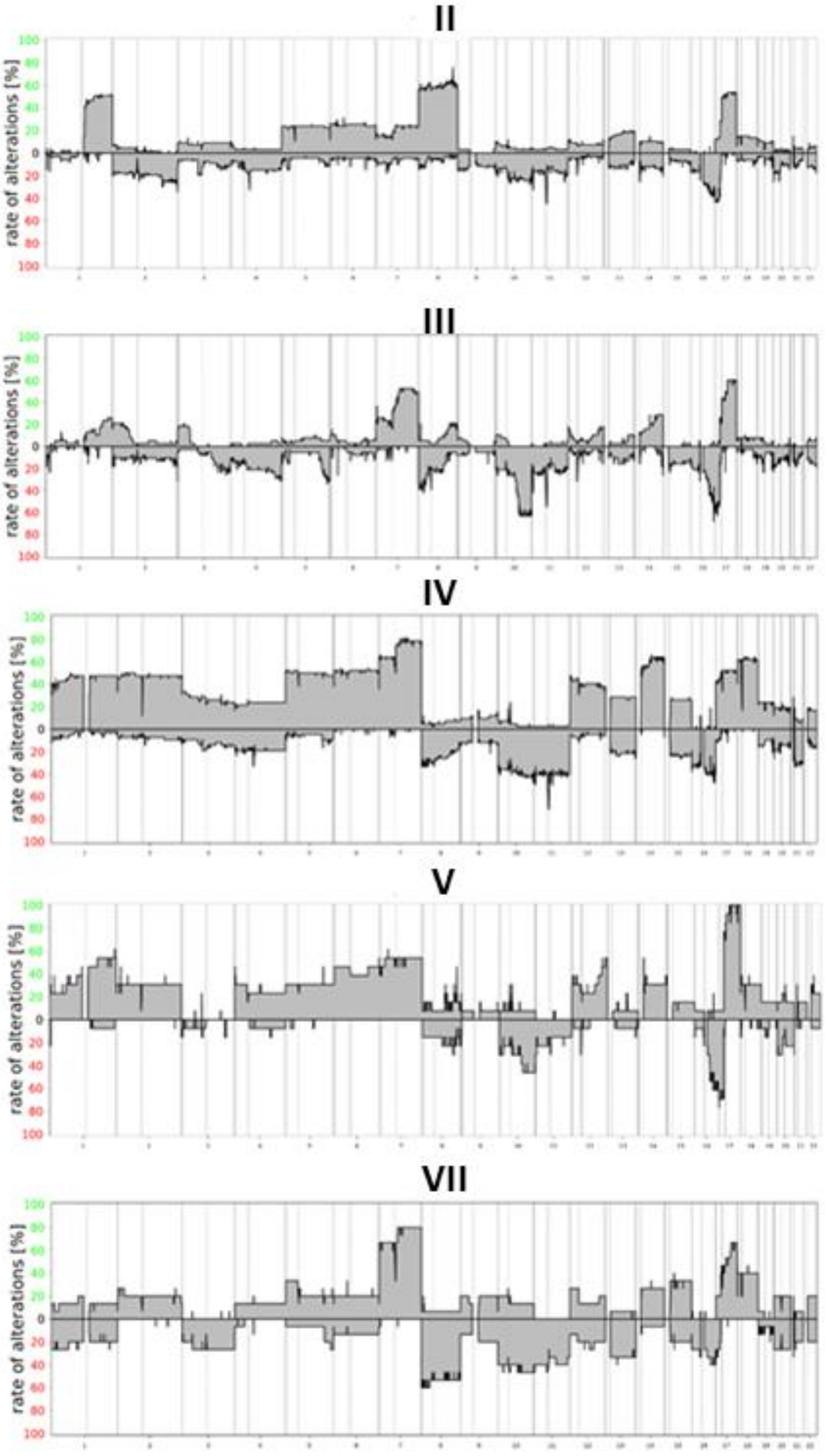


**<u>Supplementary Figure 1.</u>** CNVs profiles generated for various second-generation subgroups identified within Grp 3 MB.

**a.**

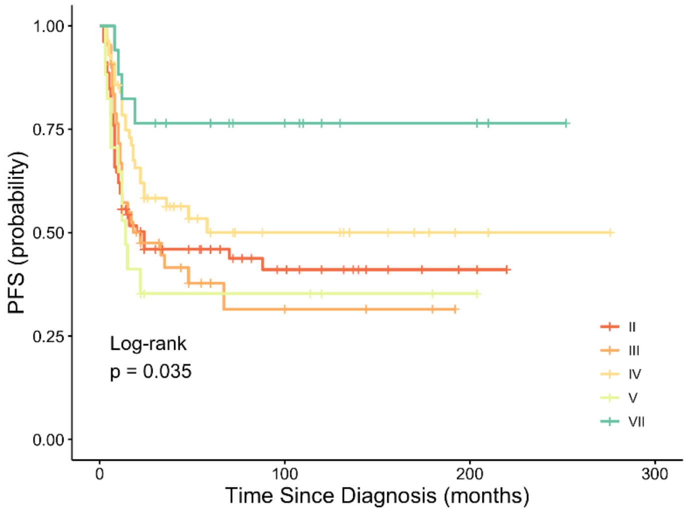


No. at Risk (# events)

| | | | |
|---|---|---|---|
| 79 (0) | 14 (44) | 4 (44) | 0 (44) |
| 43 (0) | 5 (26) | 0 (26) | 0 (26) |
| 56 (0) | 9 (26) | 2 (26) | 0 (26) |
| 17 (0) | 4 (11) | 1 (11) | 0 (11) |
| 17 (0) | 8 (4) | 3 (4) | 0 (4) |

**b.**

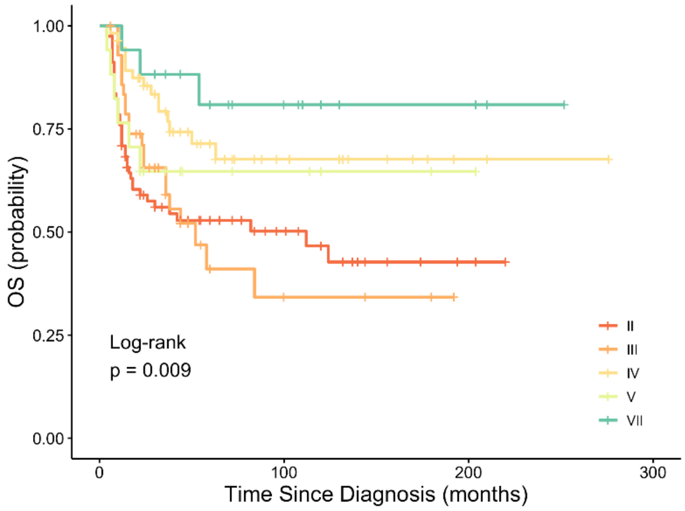


No. at Risk (# events)

| | | | |
|---|---|---|---|
| 79 (0) | 16 (37) | 4 (39) | 0 (39) |
| 43 (0) | 5 (21) | 0 (21) | 0 (21) |
| 56 (0) | 10 (15) | 2 (15) | 0 (15) |
| 17 (0) | 4 (6) | 1 (6) | 0 (6) |
| 17 (0) | 8 (3) | 3 (3) | 0 (3) |

**Supplementary Figure 2.** Survival analysis by Kaplan-Meier plotted of second-generation Grp 3 MB subgroups. Thus, subgroups IV and VII revealed significantly better PFS **(a)** and OS **(b)** as compared to subgroups II, III and V.

**Supplementary Table 1. List of genes differentially expressed in Group 3 MB with various outcomes**

| Gene | LogFC | AveExpr | P.Value | Adj.P.Val |
|---|---|---|---|---|
| *MYC* | 1,795375932 | 3,967585111 | 1,19E-07 | 0,001507289 |
| *KIRREL2* | 0,911975267 | 2,020354754 | 1,47E-07 | 0,001507289 |
| *ITPRIPL1* | 0,641901572 | 1,467529031 | 1,88E-07 | 0,001507289 |
| *DCAF4* | 0,558717404 | 1,886352803 | 1,98E-07 | 0,001507289 |
| *NPW* | 0,696787869 | 0,653871128 | 2,41E-07 | 0,001507289 |
| *CDT1* | 0,71536121 | 2,246769615 | 4,56E-07 | 0,002172801 |
| *MAB21L2* | 0,847060421 | 2,496060429 | 5,63E-07 | 0,002172801 |
| *MTHFD2* | 1,542401705 | 2,398412223 | 6,70E-07 | 0,002172801 |
| *SNORD115-5* | -2,374098718 | 5,391567474 | 6,10E-07 | 0,002172801 |
| *HSPA1B* | 1,082960737 | 4,824562514 | 7,53E-07 | 0,002172801 |
| *FASN* | 0,754443495 | 5,734571765 | 1,04E-06 | 0,002579872 |
| *SLC6A1* | -0,744523062 | 1,752718032 | 1,07E-06 | 0,002579872 |
| *ACLY* | 0,544686769 | 6,447560471 | 1,20E-06 | 0,002677319 |
| *NCAPH* | 0,693616351 | 3,098267669 | 1,30E-06 | 0,002717763 |
| *FEN1* | 0,510208962 | 2,852451586 | 1,78E-06 | 0,003491137 |
| *MRPS34* | 0,611738991 | 3,127763181 | 2,08E-06 | 0,003831095 |
| *HIST2H3D* | 1,090477366 | 4,961890544 | 2,82E-06 | 0,004901278 |
| *PITPNA* | -0,622982799 | 3,699098597 | 3,22E-06 | 0,005041655 |
| *DCTPP1* | 0,706421026 | 2,537760795 | 3,44E-06 | 0,005102363 |
| *AC010761.8* | 0,535137962 | 1,198695107 | 3,59E-06 | 0,005102363 |
| *RANBP3L* | -0,656321708 | 1,361888464 | 4,68E-06 | 0,005978534 |
| *MCM10* | 0,640108235 | 2,478406241 | 4,91E-06 | 0,005978534 |
| *NTN3* | 0,537356585 | 1,787392141 | 5,15E-06 | 0,005978534 |
| *NME1* | 0,622855031 | 2,630868396 | 7,43E-06 | 0,007878407 |
| *BZW2* | 0,695208048 | 3,541562756 | 7,58E-06 | 0,007878407 |
| *HMGA1* | 0,946584567 | 5,648547405 | 7,80E-06 | 0,007878407 |
| *RAN* | 0,555291569 | 4,113900154 | 8,40E-06 | 0,008222257 |
| *LRP1* | -0,61347828 | 4,024855163 | 9,17E-06 | 0,008553208 |
| *ANKRD44* | -0,607327808 | 2,225257375 | 9,33E-06 | 0,008553208 |
| *TMEM97* | 0,726851835 | 3,085900681 | 9,56E-06 | 0,008553208 |
| *TPI1P1* | 0,604043603 | 2,239460997 | 1,17E-05 | 0,009228367 |
| *SPOCK2* | -0,71206269 | 1,658269843 | 1,23E-05 | 0,009228367 |
| *FDFT1* | 0,73846908 | 3,274396508 | 1,25E-05 | 0,009228367 |
| *ENO1* | 0,654367469 | 6,523342646 | 1,27E-05 | 0,009228367 |
| *SIGMAR1* | 0,63258282 | 3,343658305 | 1,28E-05 | 0,009228367 |
| *SREBF2* | 0,520085427 | 5,423307501 | 1,31E-05 | 0,009228367 |
| *PHACTR2* | -0,746389549 | 2,379044022 | 1,32E-05 | 0,009228367 |
| *RCC1* | 0,657394187 | 2,836325852 | 1,39E-05 | 0,009246808 |
| *SLC25A39* | 0,570390564 | 3,844715914 | 1,53E-05 | 0,009317924 |
| *DHCR7* | 0,518034466 | 1,801278613 | 1,55E-05 | 0,009317924 |
| *WDR96* | -0,663398792 | 1,672016852 | 1,85E-05 | 0,01034211 |
| *FADS1* | 0,504890837 | 2,546491966 | 1,88E-05 | 0,010343897 |
| *DHCR24* | 0,76634286 | 2,533445429 | 1,98E-05 | 0,01048287 |
| *RCOR2* | 0,801461828 | 5,159107135 | 2,01E-05 | 0,01048287 |
| *APCDD1* | -0,992845418 | 2,645321537 | 2,09E-05 | 0,010547575 |
| *YBX1P10* | 0,56723935 | 1,692335196 | 2,09E-05 | 0,010547575 |
| *HIST2H2BF* | 0,843641944 | 6,578715975 | 2,18E-05 | 0,01062099 |
| *LDHB* | 1,074336481 | 6,394907417 | 2,20E-05 | 0,01062099 |
| *LRRC37A3* | -0,705398413 | 4,101771569 | 2,21E-05 | 0,01062099 |
| *NTM* | -0,585955903 | 1,529352643 | 2,27E-05 | 0,01062099 |
| *PYCR1* | 0,696682131 | 3,770108499 | 2,50E-05 | 0,011365902 |
| *ATP1A2* | -0,935020776 | 3,376236285 | 2,78E-05 | 0,011939696 |
| *RPL18AP3* | 0,754198295 | 2,160977885 | 2,80E-05 | 0,011939696 |
| *EIF4B* | 0,664538124 | 5,024484836 | 2,82E-05 | 0,011939696 |
| *PLAGL1* | -0,794529272 | 2,008085819 | 2,95E-05 | 0,012143417 |
| *SQLE* | 0,66981635 | 3,254327771 | 3,27E-05 | 0,013313053 |
| *TKT* | 0,684487834 | 3,316429203 | 3,32E-05 | 0,013318888 |

| | | | | |
|---|---|---|---|---|
| ***RPL14P1*** | 0,730445106 | 3,275393153 | 3,45E-05 | 0,013320995 |
| ***HIST3H2BA*** | 0,975688976 | 1,783515769 | 3,70E-05 | 0,013320995 |
| ***HIST1H1B*** | 0,939310825 | 6,378838108 | 3,76E-05 | 0,013320995 |
| ***PAICS*** | 0,739889707 | 3,973335011 | 4,05E-05 | 0,01376999 |
| ***CPNE4*** | -0,976834599 | 2,565316509 | 4,18E-05 | 0,013896114 |
| ***EPB41L1*** | -0,623552427 | 4,132323002 | 4,22E-05 | 0,013896114 |
| ***NOB1*** | 0,650269708 | 3,106466429 | 4,37E-05 | 0,014130083 |
| ***BOP1*** | 0,643004714 | 2,694499511 | 4,38E-05 | 0,014130083 |
| ***REC8*** | 0,646034834 | 4,296555839 | 4,42E-05 | 0,014130083 |
| ***EIF4BP3*** | 0,551645751 | 2,024571211 | 4,63E-05 | 0,014637247 |
| ***DKK3*** | -0,638975292 | 1,745512451 | 4,87E-05 | 0,014966716 |
| ***RNASEH2A*** | 0,575546553 | 4,654505493 | 4,92E-05 | 0,014966716 |
| ***HSPE1*** | 0,582423925 | 2,355679864 | 5,06E-05 | 0,015244515 |
| ***ETV5*** | -0,623668727 | 1,516683351 | 5,37E-05 | 0,015862279 |
| ***RP11-1336O20.2*** | 0,74609934 | 1,456489731 | 5,64E-05 | 0,016491688 |
| ***AHCY*** | 0,679771594 | 3,825068748 | 6,11E-05 | 0,016898981 |
| ***CCT3*** | 0,608155497 | 5,525056552 | 6,16E-05 | 0,016898981 |
| ***RCBTB2*** | -0,553364784 | 2,410171575 | 6,19E-05 | 0,016898981 |
| ***PBK*** | 0,731782949 | 2,997145484 | 6,20E-05 | 0,016898981 |
| ***SNHG16**** | 0,635674653 | 2,417035864 | 6,29E-05 | 0,016898981 |
| ***PTMAP2*** | 0,613878254 | 4,250471832 | 6,33E-05 | 0,016898981 |
| ***NTRK2*** | -0,690756822 | 2,158331113 | 6,37E-05 | 0,016898981 |
| ***ALYREF*** | 0,501607515 | 3,481857694 | 6,46E-05 | 0,01691754 |
| ***SNRPB*** | 0,501664075 | 3,124999623 | 6,48E-05 | 0,01691754 |
| ***COCH*** | 0,587378006 | 2,963221115 | 6,76E-05 | 0,0173455 |
| ***IL6ST*** | -0,502300923 | 3,110265139 | 7,09E-05 | 0,017832236 |
| ***CCDC136*** | -0,842361467 | 2,252735262 | 7,11E-05 | 0,017832236 |
| ***LINGO2*** | -0,741213502 | 1,825686474 | 7,32E-05 | 0,018054682 |
| ***SNORD115-21*** | -1,696587842 | 3,453661881 | 7,43E-05 | 0,018165635 |
| ***RPS7P1*** | 0,839963334 | 3,652857503 | 7,68E-05 | 0,018332598 |
| ***PCDHGC3*** | -0,551833565 | 3,359372372 | 7,69E-05 | 0,018332598 |
| ***MRPL54*** | 0,502293397 | 2,918425269 | 7,73E-05 | 0,018332598 |
| ***RPS3*** | 0,804522168 | 5,669797674 | 8,17E-05 | 0,018944263 |
| ***HSPD1*** | 0,703047993 | 6,547429872 | 8,31E-05 | 0,018969122 |
| ***ORC6*** | 0,544834692 | 2,770055586 | 8,37E-05 | 0,018969122 |
| ***ZNF117*** | -0,761554566 | 4,431085274 | 8,54E-05 | 0,018969122 |
| ***CDCA8*** | 0,567786994 | 3,280068677 | 8,66E-05 | 0,019101298 |
| ***BBS9*** | -0,617256196 | 4,542252814 | 9,05E-05 | 0,019541663 |
| ***PA2G4*** | 0,531295916 | 4,437676428 | 9,21E-05 | 0,019609178 |
| ***MIR340*** | -0,95776427 | 3,014245132 | 9,47E-05 | 0,019915713 |
| ***EIF4BP7*** | 0,505384916 | 1,366084785 | 9,48E-05 | 0,019915713 |
| ***PHB*** | 0,556984895 | 3,644697472 | 9,77E-05 | 0,020080203 |
| ***TACC3*** | 0,503172676 | 2,603033126 | 9,90E-05 | 0,020080203 |
| ***RPS16*** | 0,726511732 | 5,823448051 | 0,000100236 | 0,020080203 |
| ***UTRN*** | -0,709029932 | 3,210482433 | 0,00010039 | 0,020080203 |
| ***FAM71F2*** | -0,566695447 | 1,951700902 | 0,000101268 | 0,020080203 |
| ***RSL24D1*** | 0,566948331 | 4,182940346 | 0,000107311 | 0,020740754 |
| ***PODN*** | -0,637835965 | 1,493996564 | 0,000108814 | 0,020902306 |
| ***NDRG2*** | -0,511920773 | 2,656671134 | 0,000111959 | 0,021112191 |
| ***LHX3*** | 0,546550796 | 0,592441147 | 0,000112265 | 0,021112191 |
| ***RPS2P5*** | 0,728061776 | 1,900284377 | 0,000114482 | 0,021112191 |
| ***SLC27A2*** | 0,513358613 | 0,958723252 | 0,000115049 | 0,021112191 |
| ***ZBTB38*** | -0,50691445 | 2,098802813 | 0,000115301 | 0,021112191 |
| ***DDX21*** | 0,517881759 | 4,408650044 | 0,000121894 | 0,021630497 |
| ***FBL*** | 0,707980485 | 5,167344558 | 0,000122276 | 0,021630497 |
| ***HIST1H1E*** | 0,632204907 | 7,655891153 | 0,000124686 | 0,02168912 |
| ***PFKM*** | 0,55204908 | 5,001505548 | 0,000127771 | 0,022065393 |
| ***AURKA*** | 0,608879492 | 3,591458483 | 0,000128258 | 0,022065393 |
| ***UTP18*** | 0,506268847 | 4,312547466 | 0,000135855 | 0,023045778 |
| ***SORBS1*** | -0,537445857 | 1,655010195 | 0,000139213 | 0,023403294 |
| ***GSG1*** | 0,948010142 | 2,285424281 | 0,000147094 | 0,024055752 |

| | | | | |
|---|---|---|---|---|
| ***HIST1H2BO*** | 0,986589914 | 7,100108763 | 0,000151777 | 0,024565477 |
| ***RPL18A*** | 0,757761486 | 4,708826535 | 0,000153448 | 0,024565477 |
| ***JPH1*** | 0,54146761 | 1,229320976 | 0,000153775 | 0,024565477 |
| ***RRP9*** | 0,534449378 | 4,175097854 | 0,000161682 | 0,025567873 |
| ***RRM2*** | 0,541194166 | 3,246647218 | 0,000166389 | 0,026049067 |
| ***PTPRO*** | -0,912701336 | 5,680629162 | 0,000168624 | 0,026108601 |
| ***APRT*** | 0,704849851 | 3,136610318 | 0,000169723 | 0,026108601 |
| ***AC005154.7*** | -0,667843988 | 4,185696329 | 0,000170939 | 0,026108601 |
| ***EIF4BP6*** | 0,601797431 | 2,081671765 | 0,00017339 | 0,026253095 |
| ***PGK1*** | 0,515751649 | 5,260276204 | 0,000173562 | 0,026253095 |
| ***ZNF239*** | 0,504497162 | 2,202112681 | 0,000175354 | 0,026396719 |
| ***FAM86A*** | 0,506148535 | 3,216052071 | 0,000187958 | 0,027582048 |
| ***PCDHB17*** | -0,797645764 | 2,995132353 | 0,000189395 | 0,027582048 |
| ***CKS2*** | 0,672357374 | 4,084831547 | 0,000193348 | 0,028027389 |
| ***KCNK1*** | -0,583466851 | 1,106207291 | 0,000194696 | 0,028038717 |
| ***RAC3*** | 0,593593587 | 3,180883697 | 0,000200399 | 0,028534569 |
| ***CCDC88C*** | 0,550168438 | 3,395866778 | 0,000200492 | 0,028534569 |
| ***RPL4*** | 0,73376242 | 7,321556111 | 0,000209197 | 0,029638718 |
| ***TAF4B*** | 0,536115673 | 2,543432069 | 0,000211158 | 0,029781821 |
| ***LEPR*** | -0,500848994 | 2,227670564 | 0,000214808 | 0,03007523 |
| ***PPIAP22*** | 0,630204452 | 4,503696661 | 0,000226872 | 0,030351354 |
| ***SNORD115-25*** | -1,628107851 | 3,637888111 | 0,000235352 | 0,031225003 |
| ***TRPM3*** | -1,015883873 | 2,432296888 | 0,000238129 | 0,031460103 |
| ***RP11-466H18.1*** | 0,851908905 | 4,419660486 | 0,000239319 | 0,031484518 |
| ***RPL4P5*** | 0,522199345 | 1,464780221 | 0,000246841 | 0,031746546 |
| ***RPL5*** | 0,627012814 | 7,892681814 | 0,000250937 | 0,031746546 |
| ***RPL13AP5*** | 0,734695695 | 5,106003317 | 0,000253698 | 0,031746546 |
| ***UBE2S*** | 0,596403977 | 4,204969362 | 0,000258074 | 0,031746546 |
| ***ANKS1B*** | -0,778565014 | 3,363307617 | 0,000261148 | 0,031746546 |
| ***RFTN1*** | -0,523184396 | 2,450801558 | 0,0002621 | 0,031746546 |
| ***RPL13*** | 0,749439774 | 4,678830511 | 0,000262667 | 0,031746546 |
| ***RASGEF1C*** | -0,537843889 | 1,553381543 | 0,000267183 | 0,031746546 |
| ***EIF4EBP1*** | 0,734141797 | 3,048388574 | 0,000267935 | 0,031746546 |
| ***SLC6A15*** | 0,721726862 | 1,894772997 | 0,000268756 | 0,031746546 |
| ***PRMT1*** | 0,510353159 | 4,935223505 | 0,000269068 | 0,031746546 |
| ***IMPDH2*** | 0,656274514 | 5,989019133 | 0,000270714 | 0,031746546 |
| ***MRPL15*** | 0,549429797 | 3,735949917 | 0,000273709 | 0,031972695 |
| ***HES6*** | 0,839139797 | 5,153642061 | 0,000276816 | 0,031972695 |
| ***SPINK9*** | -0,588101194 | 2,037750927 | 0,00028044 | 0,032076402 |
| ***MEIS3P1*** | -0,601169357 | 2,078587489 | 0,000288004 | 0,032408214 |
| ***LGI1*** | -0,970251026 | 2,589041539 | 0,000313133 | 0,034179118 |
| ***ATP8A2*** | -0,85780488 | 2,139435052 | 0,000313289 | 0,034179118 |
| ***CPNE7*** | 0,976334544 | 1,421700095 | 0,000324026 | 0,03427557 |
| ***CTTNBP2*** | -1,173946787 | 2,533247686 | 0,000327484 | 0,034414702 |
| ***PCDHGA2*** | -0,804419984 | 3,193245954 | 0,000327539 | 0,034414702 |
| ***ELMO1*** | -0,545571185 | 1,887078981 | 0,000333322 | 0,034788866 |
| ***CDC20*** | 0,61780642 | 3,584806431 | 0,00033771 | 0,035013354 |
| ***SERPING1*** | -0,696899702 | 4,207702621 | 0,000349098 | 0,035720958 |
| ***CACNA2D2*** | -0,611474371 | 2,774495971 | 0,000353505 | 0,036054052 |
| ***RPS7*** | 0,670377632 | 6,788130393 | 0,000359165 | 0,036452636 |
| ***KCNA1*** | -0,670983756 | 0,877773405 | 0,000359741 | 0,036452636 |
| ***FGFR1*** | -0,696608667 | 2,787342418 | 0,00036272 | 0,03646359 |
| ***PCDHB13*** | -0,723010499 | 5,069424446 | 0,000363343 | 0,03646359 |
| ***LOXL4*** | 0,733728562 | 2,378020564 | 0,000369009 | 0,036613927 |
| ***PRDX4*** | 0,628507408 | 3,713535687 | 0,000369519 | 0,036613927 |
| ***CGNL1*** | -0,596742246 | 2,029107687 | 0,000372356 | 0,036778665 |
| ***BCAT1*** | 0,614173026 | 1,311621248 | 0,000390197 | 0,038060638 |
| ***PBX3*** | 0,648526039 | 1,762297792 | 0,000395233 | 0,038194898 |
| ***HIST1H3D*** | 0,77599335 | 6,914507655 | 0,00039807 | 0,038350678 |
| ***RPL4P4*** | 0,502451161 | 3,413852387 | 0,000402293 | 0,038520507 |
| ***HIST1H2AI*** | 0,932457427 | 6,117242204 | 0,000406564 | 0,038532979 |

| | | | | |
|---|---|---|---|---|
| ***RPS3A*** | 0,711090472 | 5,881429794 | 0,000406765 | 0,038532979 |
| ***KPNA2*** | 0,529787089 | 5,293714323 | 0,000411652 | 0,038617231 |
| ***MST4*** | 0,555110863 | 1,131396857 | 0,000425666 | 0,039085085 |
| ***GRIN2B*** | -0,571601236 | 1,808226109 | 0,000431567 | 0,039265236 |
| ***NLGN2*** | -0,500785089 | 3,268523219 | 0,000433898 | 0,039265236 |
| ***RP11-234A1.1*** | 0,561812585 | 4,852541494 | 0,000441294 | 0,03959127 |
| ***CMSS1*** | 0,527504638 | 3,647026475 | 0,000445635 | 0,039788135 |
| ***SLC44A2*** | -0,595197941 | 4,856977021 | 0,000453243 | 0,040088922 |
| ***RPL14*** | 0,645573962 | 7,384321078 | 0,000459703 | 0,040318639 |
| ***KLHL5*** | -0,570420809 | 2,565711911 | 0,000462965 | 0,040491359 |
| ***MYBL2*** | 0,614925851 | 3,508511792 | 0,000471231 | 0,040985289 |
| ***FNDC4*** | -0,549600297 | 1,856534617 | 0,000478929 | 0,041424685 |
| ***EML4*** | 0,526968691 | 4,566858534 | 0,000485739 | 0,041732818 |
| ***LDHA*** | 0,535352247 | 6,556796481 | 0,000489042 | 0,041837179 |
| ***UBE2C*** | 0,589927351 | 3,821154169 | 0,000491352 | 0,041920234 |
| ***KIAA1199*** | -0,639683175 | 3,697826466 | 0,000497582 | 0,042115037 |
| ***HIST1H2BD*** | 0,546236369 | 8,322979404 | 0,000500049 | 0,042115037 |
| ***PLXNA2*** | -0,930885584 | 3,547152141 | 0,000502156 | 0,042115037 |
| ***SMS*** | 0,578768652 | 5,985995332 | 0,000512682 | 0,042595958 |
| ***NPS*** | 0,918584845 | 1,290137111 | 0,000513767 | 0,042595958 |
| ***SNRPE*** | 0,538832346 | 4,353132302 | 0,000524067 | 0,0431254 |
| ***PPA1*** | 0,67742783 | 4,573347059 | 0,000524761 | 0,0431254 |
| ***HIST1H1C*** | 0,596410028 | 7,806687838 | 0,000534628 | 0,04347985 |
| ***RPL29*** | 0,742631743 | 4,930709552 | 0,000538369 | 0,043613493 |
| ***PDIA5*** | 0,596627107 | 3,757636491 | 0,00054045 | 0,043613493 |
| ***CCT4*** | 0,522203582 | 5,573027698 | 0,000548785 | 0,044172234 |
| ***RPL3P4*** | 0,572052963 | 3,741810172 | 0,000556126 | 0,044509237 |
| ***HIST1H2AG*** | 0,775833493 | 6,040397302 | 0,000559558 | 0,044580975 |
| ***HIST2H2AC*** | 0,588552608 | 7,746828137 | 0,000561513 | 0,044623205 |
| ***TOP1MT*** | 0,540523214 | 1,516957845 | 0,000571543 | 0,044787608 |
| ***PARM1*** | -0,531848388 | 1,832310223 | 0,000577866 | 0,045121138 |
| ***SERPINB6*** | -0,516593396 | 3,497217674 | 0,000587031 | 0,045340792 |
| ***PTGDS*** | -0,654121779 | 1,762272611 | 0,000589514 | 0,045340792 |
| ***SCD*** | 0,614141876 | 3,024189175 | 0,00059084 | 0,045340792 |
| ***RPS15A*** | 0,65898658 | 4,609055778 | 0,000591534 | 0,045340792 |
| ***RPS3AP6*** | 0,630615613 | 2,830212464 | 0,000598731 | 0,045612784 |
| ***ADAMTS7*** | -0,611580239 | 1,787999121 | 0,000605784 | 0,04603809 |
| ***RPSAP58*** | 0,534549976 | 2,413219677 | 0,00061238 | 0,046327403 |
| ***PCDHB12*** | -0,663361589 | 4,960635587 | 0,000615671 | 0,046339591 |
| ***DAO*** | -0,548166951 | 1,346196648 | 0,000651163 | 0,047748368 |
| ***RPL7A*** | 0,691811606 | 7,410522823 | 0,000653998 | 0,047844197 |
| ***PTDSS1*** | 0,509925166 | 3,160915036 | 0,000660891 | 0,048011949 |
| ***RPS5*** | 0,710264729 | 6,135151094 | 0,000677285 | 0,048527422 |
| ***DPP7*** | 0,549221829 | 3,521087728 | 0,000678995 | 0,048538844 |
| ***RPLP0P6*** | 0,611499239 | 3,199592378 | 0,000687865 | 0,049060947 |

**Supplementary Table 2. Survival analysis for genes reported as prognostic indicators in Group 3 MB**

| Author | Gene | Expression | HR PFS | P value | HR OS | P value |
|---|---|---|---|---|---|---|
| **Remke et al. (26)** | ***FSTL5*** | **Upregulated** | 0.34 | 0.55 | 0.18 | 0.67 |
| **Thompson et al. (33)** | ***VEGFA*** | **Upregulated** | 2.44 | 0.12 | 2.23 | 0.18 |
| **das Chagas et al. (8)** | ***ITPR1*** | **Downregulated** | 1.47 | 0.22 | 3.31 | 0.07 |
| **das Chagas et al. (8)** | ***RGL1*** | **Downregulated** | 3.06 | 0.08 | **5.72** | **0.02** |
| **das Chagas et al. (8)** | ***ANTXR1*** | **Downregulated** | 0.73 | 0.39 | 0.01 | 0.99 |
| **Qin et al. (24)** | ***AP1S2*** | **Upregulated** | 1.97 | 0.15 | 1.71 | 0.39 |
| **Qin et al. (24)** | ***GRM8*** | **Upregulated** | 0.67 | 0.41 | 0.93 | 0.33 |
| **Mutlu et al. (19)** | ***MALAT1*** | **Upregulated** | 0.31 | 0.58 | 0.97 | 0.32 |
| **Mutlu et al. (19)** | ***SNHG16**** | **Upregulated** | **8.98** | **0.01** | **13.22** | **0.01** |
| **Riemondy et al. (27)** | ***IMPG2*** | **Upregulated** | 0.76 | 0.38 | 0.25 | 0.61 |
| **Delaidelli et al. (9)** | ***TPD52*** | **Upregulated** | 1.73 | 0.19 | **3.57** | **0.03** |
| **Luo et al. (18)** | ***ZNF124*** | **Upregulated** | 0.61 | 0.43 | 0.22 | 0.74 |

***Included in the current DEG gene list (see Suppl. Table 1)**

**Supplementary Table 3. Correlation between expression *MYC* and other prognostically significant genes**

| Genes correlation | Correlation coefficient | P value |
|---|---|---|
| ***MYC vs. KIRREL2*** | **0.074** | **0.37** |
| ***MYC vs. ITPRIPL1*** | **0.388** | **< 0.01** |
| ***MYC vs.DCAF4*** | **0.615** | **< 0.01** |
| ***MYC vs. NPW*** | **0.649** | **< 0.01** |
| ***MYC vs. CDT1*** | **0.371** | **< 0.01** |
| ***MYC vs. MAB21L2*** | **0.555** | **< 0.01** |

**Supplementary Table 4. Genes correlated with *KIRREL2* in Group 3 medulloblastoma**

| Gene | Correlation coefficient | P value | Location |
|---|---|---|---|
| ***NPHS1*** | 0.657314167383812 | < 0.01 | 19q13.12 |
| ***GSG1*** | 0.654477717945629 | < 0.01 | 12p13.1 |
| ***CABP2*** | 0.636438048630248 | < 0.01 | 11q13.2 |
| ***SORCS2*** | 0.607556202839938 | < 0.01 | 4p16.1 |
| ***FAM57B*** | 0.551225762800319 | < 0.01 | 16p11.2 |
| ***TMCC2*** | 0.566931501428839 | < 0.01 | 1q32.1 |
| ***CKMT1A*** | 0.563888703781324 | < 0.01 | 15q15.3 |
| ***HEBP1*** | 0.560451771049214 | < 0.01 | 12p13.1 |
| ***CCDC85C*** | 0.556861199000481 | < 0.01 | 14q32.2 |
| ***NTN3*** | 0.554624513450379 | < 0.01 | 16p13.3 |
| ***NBEAL2*** | 0.55010124858805 | < 0.01 | 3p21.31 |
| ***EML4*** | 0.542463504423775 | < 0.01 | 2p21 |
| ***SHROOM2*** | 0.541922701283555 | < 0.01 | Xp22.2 |
| ***CLN6*** | 0.542808399036347 | < 0.01 | 15q23 |
| ***RPUSD3*** | 0.53414495964933 | < 0.01 | 3p25.3 |
| ***EEF2K*** | 0.510876975978882 | < 0.01 | 16p12.2 |
| ***PDIA5*** | 0.530708006789616 | < 0.01 | 3q21.1 |
| ***GPD1L*** | 0.530729358039451 | < 0.01 | 3p22.3 |
| ***PALMD*** | 0.528565433173668 | < 0.01 | 1p21.2 |
| ***CD320*** | 0.527359346194482 | < 0.01 | 19q.13.2 |
| ***SCLY*** | 0.520445168954163 | < 0.01 | 2q37.3 |
| ***CDK5R2*** | 0.516250239724747 | < 0.01 | 2q35 |
| ***ARMC6*** | 0.511853551325067 | < 0.01 | 19p13.11 |
| ***SARS2*** | 0.512101910833845 | < 0.01 | 19q13.2 |
| ***PSAPL1*** | 0.509223751907429 | < 0.01 | 4p16.1 |
| ***RPP25*** | 0.507148003716843 | < 0.01 | 15q24.2 |
| ***FEZF2*** | 0.507238392393008 | < 0.01 | 3p14.2 |
| ***QTRTD1*** | 0.501592211601122 | < 0.01 | 3q13.31 |
| ***CERS4*** | 0.500676445636955 | < 0.01 | 19p13.2 |
| ***ANKDD1A*** | 0.476540509361738 | < 0.01 | 15q22.31 |
| ***PNO1*** | 0.476066429623778 | < 0.01 | 2p14 |
| ***CRYBG3*** | 0.498236522538543 | < 0.01 | 3q11.2 |
| ***TULP1*** | 0.497931608818322 | < 0.01 | 6p21.31 |
| ***LHX3*** | 0.492349431084144 | < 0.01 | 9q34.3 |
| ***C2ORF71*** | 0.490777755062613 | < 0.01 | 2p23.3 |
| ***MATK*** | 0.490268403977136 | < 0.01 | 19p13.3 |
| ***GALT*** | 0.488246383743145 | < 0.01 | 9p13.3 |
| ***ISOC1*** | 0.488260791409927 | < 0.01 | 5q23.3 |
| ***CACNA1B*** | 0.484770833607915 | < 0.01 | 9q34.3 |
| ***KLF16*** | 0.483712130914893 | < 0.01 | 19p13.3 |
| ***RCOR1*** | 0.481374181030577 | < 0.01 | 14q32.31 |
| ***RD3*** | 0.479626446494979 | < 0.01 | 1q32.1 |
| ***TBC1D8*** | 0.477878598852958 | < 0.01 | 2q11.2 |
| ***NCLN*** | 0.475413368247615 | < 0.01 | 19p13.3 |
| ***TIMM50*** | 0.474929999490694 | < 0.01 | 19q13.2 |
| ***PNPLA3*** | 0.474942085756096 | < 0.01 | 22q13.2 |
| ***DUSP7*** | 0.473936653091882 | < 0.01 | 3p21.31 |
| ***LRP5L*** | 0.469291197140299 | < 0.01 | 22q11.23 |
| ***MRPS25*** | 0.467485932716633 | < 0.01 | 3p25.1 |
| ***NAGPA*** | 0.465777958360065 | < 0.01 | 16p13.3 |
| ***RIMBP2*** | 0.462278342832807 | < 0.01 | 12q24.33 |
| ***RHBDL3*** | 0.462572656384838 | < 0.01 | 17q11.2 |
| ***KLHL15*** | 0.462327966762969 | < 0.01 | Xp22.11 |
| ***PPM1N*** | 0.460537468910319 | < 0.01 | 19q13.32 |
| ***QSOX2*** | 0.458926674743014 | < 0.01 | 9q34.3 |
| ***ABCG4*** | 0.45840156913308 | < 0.01 | 11q23.3 |
| ***CYP27B1*** | 0.457130915172943 | < 0.01 | 12q14.1 |
| ***TTC7B*** | 0.457158909996273 | < 0.01 | 14q32.11 |
| ***MRPS9*** | 0.456543390436614 | < 0.01 | 2q12.1 |
| ***TMEM201*** | 0.455431904331122 | < 0.01 | 1p36.22 |

**Supplementary Table 5. Signaling pathways associated with *KIRREL2*-correlated genes**

| Pathways (Gene Ontology) | Go Path | Associated Genes Found |
|---|---|---|
| **Ligase activity** | 16874; 16875 | *WARS2, FARS2, LARS2, RIMKLA, SARS2, QARS, XRCC1, TRIM25, MARS2* |
| **tRNA metabolic process** | 6399; 6418 | *RPUSD3, CDK5RAP1, RPP25, SARS2, QARS, QTRTD1, GTPBP3, RPUSD4* |
| **Mitochondrial translation** | 32543; 44429 | *MRPL10, LARS2, MRPL4, CDK5RAP1, MRPS25, MRPS9, DAP3* |
| **Cellular protein complex** | 32984; 43624 | *KIF19, MRPL10, RLTPR, MRPL4, MRPS25, MRPS9, DAP3* |
| **Translation elongation** | 6412; 6414 | *FARS2, EIF2D, EEF2K, LARS2, TACO1, SARS2, QARS, DAP3, MARS2, MRPS9* |
| **Neurogenesis** | 22008; 48699 | *CSMD3, CRX, LPPR5, EML1, INPP5J, DISC1, EEF2K, HDAC1, TLX2, C2ORF71* |
| **Synapse part; presynapse** | 44456; 98973 | *RAB40B, KPTN, DISC1, EEF2K, ASIC2, ROR1, PALMD, PVRL1, RAB3A, STX3, SYP, TULP1* |

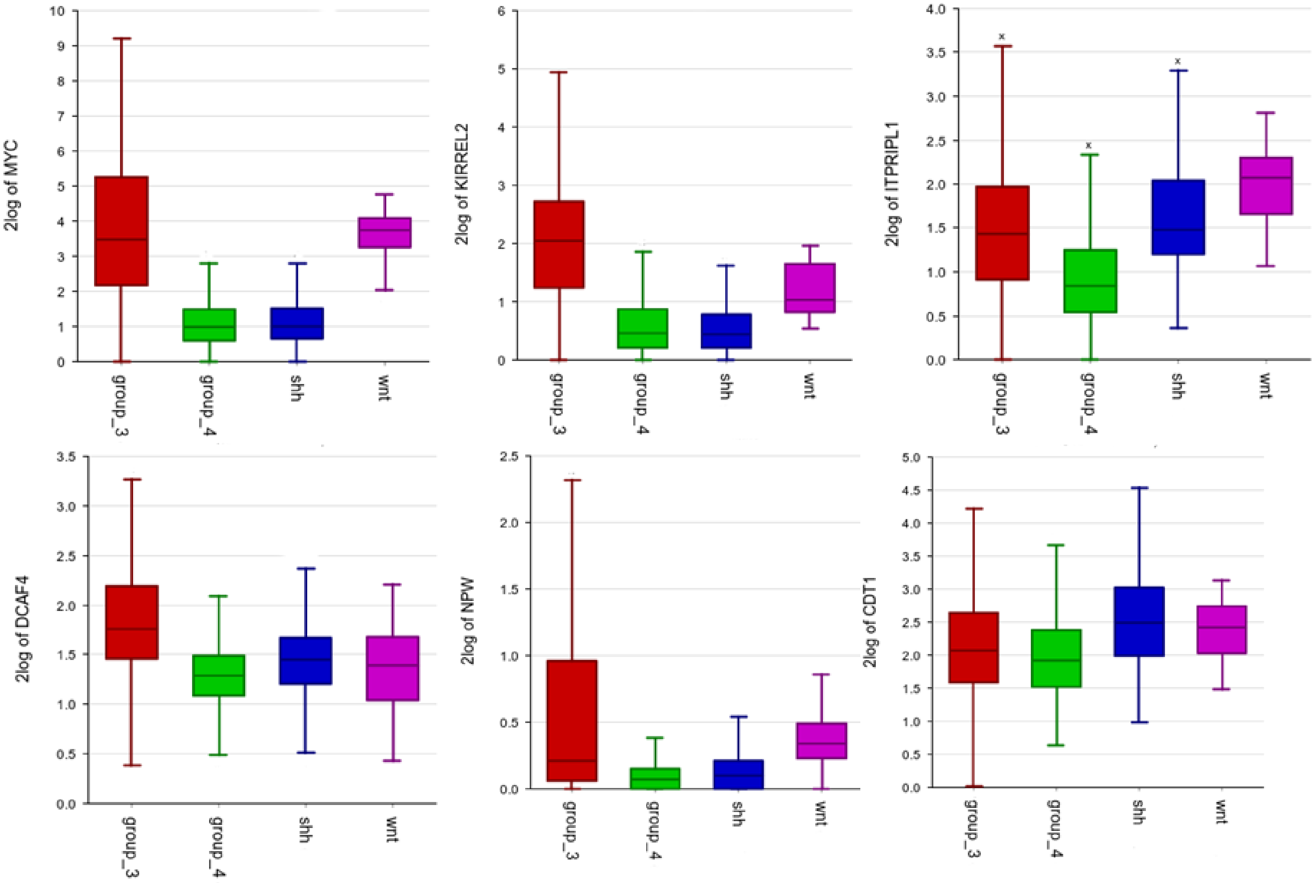


**Supplementary Figure 3.** Expression levels of top 6 prognostically relevant genes in four molecular MB groups. Expression of *KIRREL2* is significantly higher in Grp 3 MB (red box plots) as compared to Grp 4 MB (green boxplots), SHH-MB (blue boxplots) and WNT (violet boxplots). Five other genes (*MYC*, *ITPRIPL1*, *DCAF4*, *NPW*, *CDT1*) disclosed less significant differences between these canonical MB molecular groups.

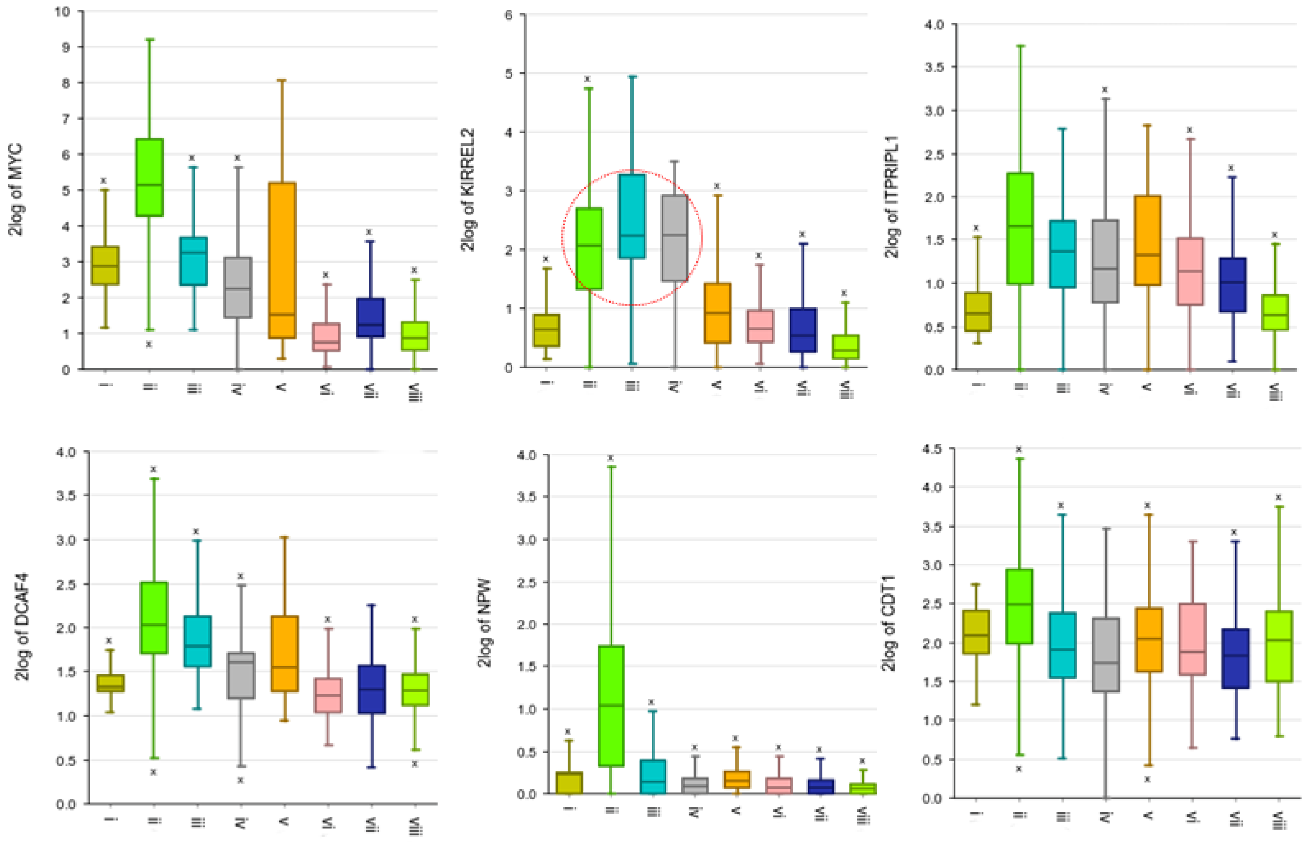


**<u>Supplementary Figure 4.</u>** Expression levels of top prognostically relevant genes in eight second generation MB subgroups. *KIRREL2* expression is quite similar in main Grp 3 MB "second generation" II, III and IV subgroups which composed 85% of Grp 3 MB in the current series. Five other genes (*MYC, ITPRIPL1, DCAF4, NPW, CDT1*) disclosed higher expression levels for second generation subgroup II mostly.

**Supplementary Table 6. Methylation levels of CpGs within *KIRREL2* promoter region.**

| CpG | CpG_Loci | Targ_Vals | Other_Vals | Meth_Var | Intercept | F | P_val | q_val |
|---|---|---|---|---|---|---|---|---|
| **cg15509065** | **chr19:36346314-36346315** | **0,298485906** | **0,417788437** | **-0,119302531** | **0,417788437** | **20,67923065** | **1,03E-05** | **0,001486624** |
| cg22339278 | chr19:36347276-36347277 | 0,068848688 | 0,075244311 | -0,006395623 | 0,075244311 | 3,960102938 | 0,048194013 | 0,146734342 |
| **cg21057435** | **chr19:36347443-36347444** | **0,08760081** | **0,122478427** | **-0,034877617** | **0,122478427** | **11,97195017** | **0,000682828** | **0,011899206** |
| cg20254353 | chr19:36347626-36347627 | 0,05794818 | 0,068181194 | -0,010233013 | 0,068181194 | 2,142610778 | 0,145104518 | 0,271018526 |
| cg17975002 | chr19:36347691-36347692 | 0,047554572 | 0,049376683 | -0,001822112 | 0,049376683 | 0,036335545 | 0,849051669 | 0,61290794 |
| cg25921512 | chr19:36347698-36347699 | 0,015388262 | 0,020707048 | -0,005318786 | 0,020707048 | 0,568690323 | 0,451823101 | 0,473457806 |
| cg00519185 | chr19:36347907-36347908 | 0,114594234 | 0,134817461 | -0,020223227 | 0,134817461 | 5,704006729 | 0,018022403 | 0,082140507 |
| cg09479015 | chr19:36347919-36347920 | 0,089165846 | 0,1066112 | -0,017445354 | 0,1066112 | 4,358352274 | 0,038318927 | 0,128548673 |
| cg06945936 | chr19:36348034-36348035 | 0,029522608 | 0,043697478 | -0,01417487 | 0,043697478 | 2,981284224 | 0,086048985 | 0,203940335 |
| **cg23087300** | **chr19:36348415-36348416** | **0,249754384** | **0,42432827** | **-0,174573886** | **0,42432827** | **46,50492802** | **1,53E-10** | **1,12E-05** |
| cg05657800 | chr19:36350479-36350480 | 0,69834912 | 0,718539811 | -0,02019069 | 0,718539811 | 1,864033232 | 0,173963981 | 0,297977727 |
| cg08141172 | chr19:36351774-36351775 | 0,835946855 | 0,850453594 | -0,014506739 | 0,850453594 | 0,734309187 | 0,392696322 | 0,444402284 |
| cg12183993 | chr19:36352005-36352006 | 0,72936527 | 0,72714277 | 0,0022225 | 0,72714277 | 0,010601704 | 0,918112446 | 0,630357632 |
| cg10786645 | chr19:36352376-36352377 | 0,755970071 | 0,713781148 | 0,042188923 | 0,713781148 | 2,036063279 | 0,155440814 | 0,28113189 |
| cg01588826 | chr19:36352459-36352460 | 0,785254616 | 0,762762836 | 0,02249178 | 0,762762836 | 2,986299356 | 0,085787715 | 0,203600242 |
| cg27035708 | chr19:36357115-36357116 | 0,785143958 | 0,775991 | 0,009152958 | 0,775991 | 0,645542662 | 0,422832794 | 0,459560158 |
| cg22467594 | chr19:36357700-36357701 | 0,835865283 | 0,817243509 | 0,018621774 | 0,817243509 | 3,002378711 | 0,08495586 | 0,202489736 |

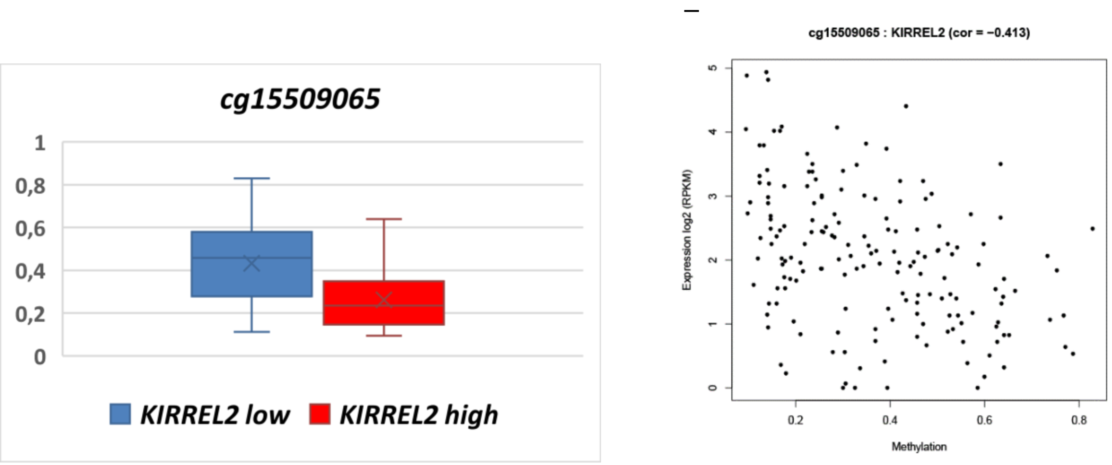


**a.**

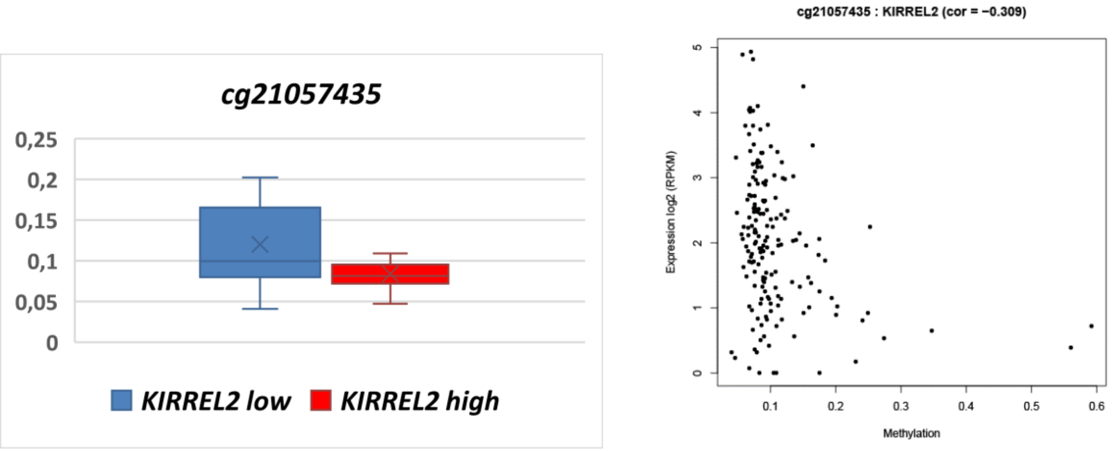


**b.**

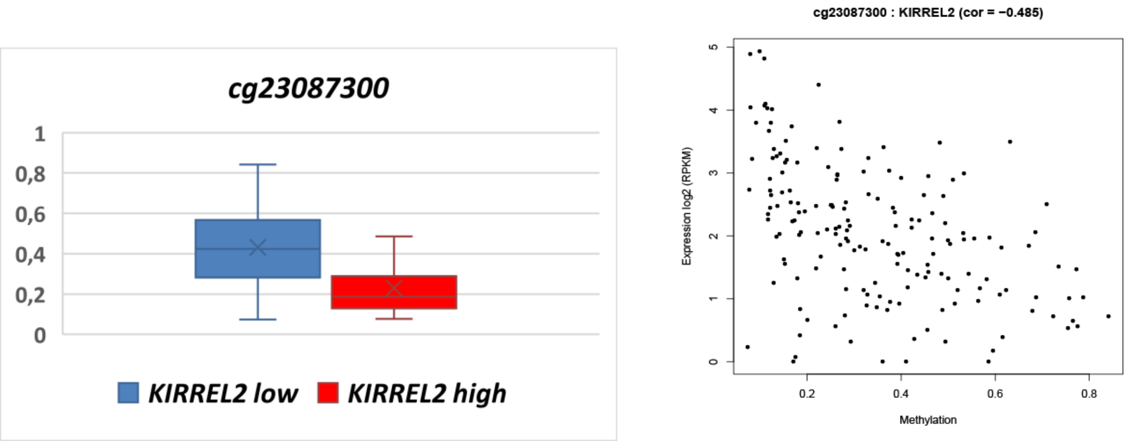


**c.**

**Supplementary Figure 5.** Methylation levels of three CpGs (cg15509065 **(a)**, cg21057435 **(b)**, cg23087300 **(c)**) within *KIRREL2* promoter region were lower in Grp 3 MB with higher gene expression and, also, negative correlations were identified between epigenetic and transcriptome data (correlation coefficients r = - 0.413; - 0.309; and – 0.485, respectively; all $p < 0.01$).

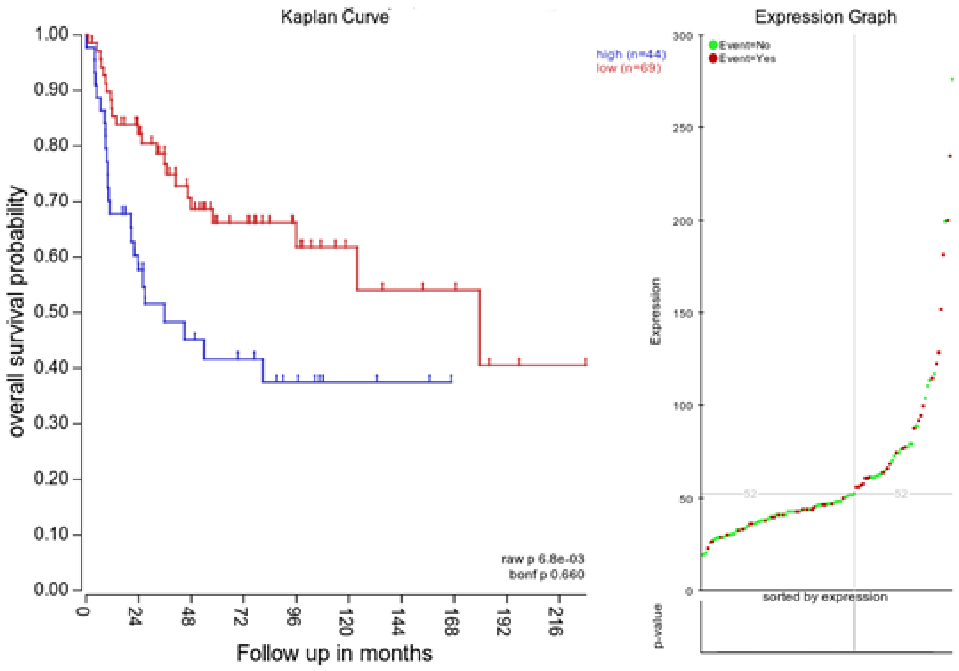


**a.**

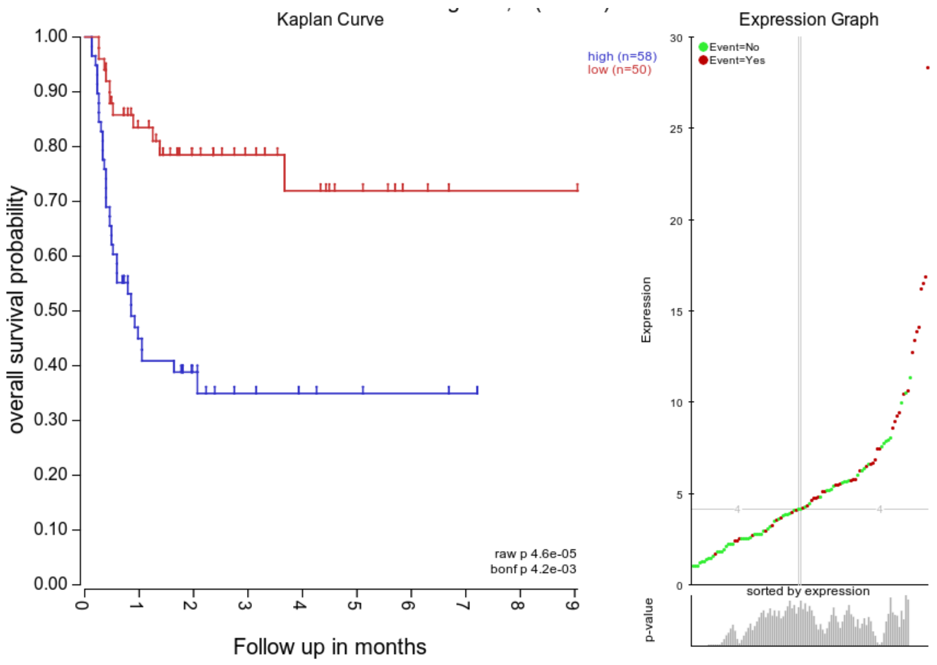


**b.**

**Supplementary Figure 6.** Survival analysis of Affymetrix gene expression profiling data generated for two independent sets of Grp 3 MB (**a.** Cavalli et al [3]; n = 113; log-rank; p < 0.01) and (**b.** Kool et al [15]; n = 108; log rank; p < 0.01) showed unfavourable overall survival for Grp 3 MB with high levels of *KIRREL2* expression (blue lines).
